# Association of COVID-19 Vaccinations With Intensive Care Unit Admissions and Outcome of Critically Ill Patients With COVID-19 Pneumonia in Lombardy, Italy

**DOI:** 10.1001/jamanetworkopen.2022.38871

**Published:** 2022-10-27

**Authors:** Giacomo Grasselli, Alberto Zanella, Eleonora Carlesso, Gaetano Florio, Arif Canakoglu, Giacomo Bellani, Nicola Bottino, Luca Cabrini, Gian Paolo Castelli, Emanuele Catena, Maurizio Cecconi, Danilo Cereda, Davide Chiumello, Andrea Forastieri, Giuseppe Foti, Marco Gemma, Riccardo Giudici, Lorenzo Grazioli, Andrea Lombardo, Ferdinando Luca Lorini, Fabiana Madotto, Alberto Mantovani, Giovanni Mistraletti, Francesco Mojoli, Silvia Mongodi, Gianpaola Monti, Stefano Muttini, Simone Piva, Alessandro Protti, Frank Rasulo, Anna Mara Scandroglio, Paolo Severgnini, Enrico Storti, Roberto Fumagalli, Antonio Pesenti

**Affiliations:** 1 Dipartimento di Anestesia, Rianimazione ed Emergenza-Urgenza, Fondazione IRCCS Ca’ Granda Ospedale Maggiore Policlinico, Milan, Italy; 2 Department of Pathophysiology and Transplantation, University of Milan, Milan, Italy; 3 Department of Anesthesia and Intensive Care Medicine, ASST Monza Ospedale San Gerardo, Monza, Italy; 4 School of Medicine and Surgery, University of Milano-Bicocca, Monza, Italy; 5 Azienda Ospedaliera Ospedale di Circolo e Fondazione Macchi, Varese, Italy; 6 Università degli Studi dell'Insubria, Varese, Italy; 7 Dipartimento di Anestesia e Rianimazione, ASST Mantova Ospedale Carlo Poma, Mantova Italy; 8 Department of Anesthesiology and Intensive Care, ASST Fatebenefratelli Sacco Luigi Sacco Hospital, Polo Universitario, Milan, Italy; 9 Department of Anaesthesia and Intensive Care Medicine, IRCCS Humanitas Clinical and Research Centre, Rozzano, Italy; 10 Humanitas University, Pieve Emanuele, Italy; 11 Directorate General for Health, Lombardy Region, Milano, Italy; 12 Department of Anesthesia and Intensive Care, San Paolo Hospital, Milano, Italy; 13 Department of Health Sciences, University of Milan, Milano, Italy; 14 Dipartimento di Anestesia e Rianimazione ASST Lecco Ospedale di Lecco, Lecco, Italy; 15 Terapia Intensiva–Neuroanestesia e Rianimazione. Fondazione IRCCS Istituto Neurologico Carlo Besta, Milano, Italy; 16 Dipartimento di Anestesia e Rianimazione, ASST Grande Ospedale Metropolitano Niguarda, Milan, Italy; 17 Department of Anaesthesia and Intensive Care, ASST Papa Giovanni XXIII, Bergamo, Italy; 18 Dipartimento Di Emergenza, Rianimazione, Anestesia–UO Anestesia e Rianimazione 2–ASST Lariana Ospedale Sant'Anna, Como, Italy; 19 Department of Anesthesia and critical care, ASST Ovest Milanese Ospedale Nuovo di Legnano, Legnano, Italy; 20 Anestesia e Rianimazione 1, Fondazione IRCCS Policlinico San Matteo, Pavia, Italy; 21 Dipartimento di Scienze Clinico-Chirurgiche Diagnostiche e Pediatriche, Università degli Studi di Pavia, Pavia, Italy; 22 SC Anestesia e Rianimazione II, Ospedale San Carlo Borromeo, ASST Santi Paolo e Carlo–Polo Universitario, Milano, Italy; 23 Department of Anesthesia, Critical Care and Emergency, Spedali Civili University Hospital, Brescia, Italy; 24 Department of Medical and Surgical Specialties, Radiological Sciences and Public Health, University of Brescia, Brescia, Italy; 25 Department of Anesthesia and Intensive Care, IRCCS San Raffaele Scientific Institute, Milano, Italy; 26 Dipartimento di Anestesia e Rianimazione ASST Cremona Ospedale di Cremona, Cremona, Italy

## Abstract

**Importance:**

Data on the association of COVID-19 vaccination with intensive care unit (ICU) admission and outcomes of patients with SARS-CoV-2–related pneumonia are scarce.

**Objective:**

To evaluate whether COVID-19 vaccination is associated with preventing ICU admission for COVID-19 pneumonia and to compare baseline characteristics and outcomes of vaccinated and unvaccinated patients admitted to an ICU.

**Design, Setting, and Participants:**

This retrospective cohort study on regional data sets reports: (1) daily number of administered vaccines and (2) data of all consecutive patients admitted to an ICU in Lombardy, Italy, from August 1 to December 15, 2021 (Delta variant predominant). Vaccinated patients received either mRNA vaccines (BNT162b2 or mRNA-1273) or adenoviral vector vaccines (ChAdOx1-S or Ad26.COV2). Incident rate ratios (IRRs) were computed from August 1, 2021, to January 31, 2022; ICU and baseline characteristics and outcomes of vaccinated and unvaccinated patients admitted to an ICU were analyzed from August 1 to December 15, 2021.

**Exposures:**

COVID-19 vaccination status (no vaccination, mRNA vaccine, adenoviral vector vaccine).

**Main Outcomes and Measures:**

The incidence IRR of ICU admission was evaluated, comparing vaccinated people with unvaccinated, adjusted for age and sex. The baseline characteristics at ICU admission of vaccinated and unvaccinated patients were investigated. The association between vaccination status at ICU admission and mortality at ICU and hospital discharge were also studied, adjusting for possible confounders.

**Results:**

Among the 10 107 674 inhabitants of Lombardy, Italy, at the time of this study, the median [IQR] age was 48 [28-64] years and 5 154 914 (51.0%) were female. Of the 7 863 417 individuals who were vaccinated (median [IQR] age: 53 [33-68] years; 4 010 343 [51.4%] female), 6 251 417 (79.5%) received an mRNA vaccine, 550 439 (7.0%) received an adenoviral vector vaccine, and 1 061 561 (13.5%) received a mix of vaccines and 4 497 875 (57.2%) were boosted. Compared with unvaccinated people, IRR of individuals who received an mRNA vaccine within 120 days from the last dose was 0.03 (95% CI, 0.03-0.04; *P* < .001), whereas IRR of individuals who received an adenoviral vector vaccine after 120 days was 0.21 (95% CI, 0.19-0.24; *P* < .001). There were 553 patients admitted to an ICU for COVID-19 pneumonia during the study period: 139 patients (25.1%) were vaccinated and 414 (74.9%) were unvaccinated. Compared with unvaccinated patients, vaccinated patients were older (median [IQR]: 72 [66-76] vs 60 [51-69] years; *P* < .001), primarily male individuals (110 patients [79.1%] vs 252 patients [60.9%]; *P* < .001), with more comorbidities (median [IQR]: 2 [1-3] vs 0 [0-1] comorbidities; *P* < .001) and had higher ratio of arterial partial pressure of oxygen (Pao_2_) and fraction of inspiratory oxygen (FiO_2_) at ICU admission (median [IQR]: 138 [100-180] vs 120 [90-158] mm Hg; *P* = .007). Factors associated with ICU and hospital mortality were higher age, premorbid heart disease, lower Pao_2_/FiO_2_ at ICU admission, and female sex (this factor only for ICU mortality). ICU and hospital mortality were similar between vaccinated and unvaccinated patients.

**Conclusions and Relevance:**

In this cohort study, mRNA and adenoviral vector vaccines were associated with significantly lower risk of ICU admission for COVID-19 pneumonia. ICU and hospital mortality were not associated with vaccinated status. These findings suggest a substantial reduction of the risk of developing COVID-19–related severe acute respiratory failure requiring ICU admission among vaccinated people.

## Key Points

**Question:**

Does COVID-19 vaccination prevent intensive care unit (ICU) admission for COVID-19 pneumonia and improve patient outcomes?

**Findings:**

In this cohort study of more than 10 million people, vaccines based on mRNA technology or adenoviral vectors significantly decreased the risk of ICU admission for COVID-19 pneumonia. ICU and hospital mortality, adjusted for age, heart disease and Pao_2_/FiO_2_ at ICU admission, were similar between vaccinated and unvaccinated patients.

**Meaning:**

COVID-19 vaccines were associated with a lower ICU admission for COVID-19 pneumonia, while no significant association was detected with ICU and hospital mortality.

## Teaser

This cohort study investigates whether COVID-19 vaccination is associated with lower risk of intensive care unit (ICU) admission for COVID-19 pneumonia and compares baseline characteristics and outcomes of vaccinated vs unvaccinated patients admitted to an ICU.

## Introduction

Several vaccines have been approved for emergency use as a response to the COVID-19 pandemic.^1,2,3,4,5,6,7,8,9,10^ Growing evidence supports the safety and efficacy of vaccination against severe acute respiratory syndrome coronavirus 2 (SARS-CoV-2) infection to prevent hospitalization and death related to COVID-19.^1,2,3,4,5,6,7,8,9^ However, precise analyses of the preventing role of vaccination on intensive care unit (ICU) admission for severe COVID-19 and a detailed description of vaccinated COVID-19 patients who are critically ill are still lacking.^11,12,13,14^

Two multicenter studies conducted in Spain and Switzerland reported that fully vaccinated patients with COVID-19 admitted to an ICU had more comorbidities, greater exposure to immunosuppressive drugs, lower ICU length of stay but similar ICU mortality compared with unvaccinated patients.^15,16^ However, in both studies, the number of vaccinated and unvaccinated patients admitted during the Alpha and Delta variant waves may have been unbalanced.

In Lombardy, a region in Northern Italy, the vaccination campaign started on December 27, 2020, and initially targeted health care personnel, people at least 80 years of age, and individuals with frailties (eFigure 1, eTable 1 in Supplement 1). Two vaccines based on mRNA technology (BNT162b2 [Pfizer-BioNTech] and mRNA-1273 [Moderna]) and 2 vaccines using adenoviral vectors (ChADOx1-S [AstraZeneca] and Ad26.COV2 [Johnson & Johnson]) have been approved. As of February 1, 2022, 89% of Lombardy’s population aged 12 years or older has been fully vaccinated, mainly with mRNA vaccines. Despite the intensive vaccination campaign, 2 pandemic waves developed in March and November 2021, and many COVID-19 patients who were critically ill, both vaccinated and unvaccinated, were admitted to one of Lombardy’s COVID-19 ICUs. The aims of the present study are to (1) determine the role of vaccination in preventing ICU admission for COVID-19 pneumonia and (2) compare baseline characteristics and outcomes of vaccinated and unvaccinated patients admitted to an ICU, focusing on patients admitted during Fall 2021, when the Delta variant was predominant.

## Methods

The Lombardy Regional Health Service approved our request to perform a retrospective cohort study to analyze (using a regional platform [Data-as-a-Service]) official data sets of citizens who had contact with the regional health care system. Italian National Health Service provides universal coverage and all provided health services are collected in the regional health care administrative databases. This cohort study used deidentified data and did not constitute human participant research; thus, the Fondazione IRCCS Ca’ Granda Ospedale Maggiore Policlinico institutional review board waived the need for approval and informed consent. The study followed the best practices described in the Strengthening the Reporting of Observational Studies in Epidemiology (STROBE) reporting guideline.^17^

Three different data sets were used in our analyses: (1) the COVID-19 vaccination registry, which collected daily data from December 27, 2020 (beginning of the Italian vaccination campaign), of the total number of citizens and individuals who received a COVID-19 vaccination, aggregating these numbers for: sex, 5-year age classes (individuals older than 91 years are collected together), type of vaccine, and number of doses; (2) the COVID-19 ICU data set, which contains anonymized demographic and clinical data of patients admitted to one of the Lombardy ICUs after August 1, 2020, with SARS-CoV-2 infection confirmed by at least 1 positive result of a real-time reverse transcriptase–polymerase chain reaction assay of nasal and pharyngeal swabs; the Lombardy Regional Health Service created this data set by integrating 7 clinical and administrative databases; the COVID-19 ICU data set includes data on: age and sex, comorbidities (hypertension, hypercholesterolemia, type 2 diabetes, heart disease, malignant neoplasms, chronic obstructive pulmonary disease [COPD], chronic kidney disease [CKD], liver disease, other) (eTable 2 in Supplement 1), immunodepression and chronic immunosuppressive treatment (eTable 3 in Supplement 1), vaccination status of the patient, clinical data on hospital and ICU admission (diagnosis of COVID-19 pneumonia, dates of diagnosis, hospitalization, ICU admission, and discharge, and ICU and hospital outcomes), and daily clinical parameters during the ICU stay (ventilatory support, fraction of inspiratory oxygen [FiO_2_], arterial partial pressure of oxygen [Pao_2_], Pao_2_/FiO_2_, positive end-expiratory pressure [PEEP], and use of prone positioning and extracorporeal membrane oxygenation [ECMO]), information on SARS-CoV-2 infections, also previous to the ICU admission; and (3) the COVID-19 registry, which reports the daily number of SARS-CoV-2 infections as well as hospitalizations and ICU admissions for COVID-19 from February 24, 2020.

Data from the 3 COVID-19 data sets were extracted on February 28, 2022. Exclusion criteria were ICU admission for reasons other than pneumonia and a previous diagnosis of SARS-CoV-2 infection. Individuals who received any different combination of vaccines were also excluded.

**Figure 1.**
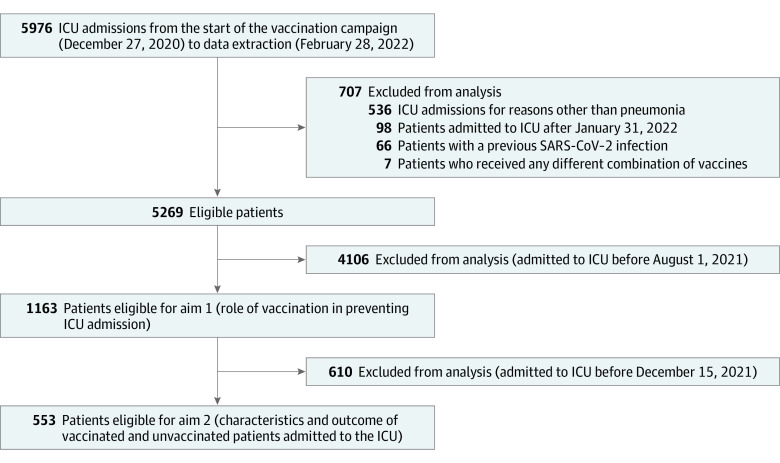
Study Flow Diagram ICU indicates intensive care unit.

To assess the role of vaccination in preventing ICU admission for COVID-19 pneumonia (aim 1), we included patients admitted to an ICU after August 1, 2021, and before January 31, 2022 (only 4 vaccinated patients were admitted to an ICU before August 1). To compare baseline characteristics and outcomes of vaccinated and unvaccinated patients admitted to an ICU (aim 2), we included patients admitted to an ICU after August 1, 2021, and before December 15, 2021, because the Delta variant was predominant in this period (Figure 1; eFigure 11 and eFigure 13 in Supplement 1). The last follow-up for hospital mortality was August 31, 2022.

### Definitions

In the present work, individuals were considered vaccinated 14 days after the second dose of BNT162b2, mRNA-1273, ChADOx1-S, or the first dose of Ad26.COV2. Vaccinated individuals who received an additional dose of vaccine were considered boosted 14 days after the administration.

Vaccinated individuals were divided into 2 groups: (1) mRNA vaccines (mRNA), if they received BNT162b2 and/or mRNA-1273 vaccines, or (2) adenoviral vector vaccines (AdV), if they received ChADOx1-S and/or Ad26.COV2 vaccines. Individuals who received any other combination of vaccines (mix), including those boosted with a different vaccine were excluded from the analysis. Individuals previously infected by SARS-CoV-2 were also excluded (Figure 1). In order to describe the clinical course of patients admitted to an ICU for COVID-19 pneumonia, we considered in the analysis the following clinical outcomes: mortality at ICU discharge, mortality at hospital discharge, length of stay in the ICU and hospital (defined as days between the dates of admission and discharge in ICU or hospital), invasive mechanical ventilation and rescue therapies use (ECMO, prone position) during ICU stay.

### Statistical Analysis

#### Association of Vaccination With ICU Admission

The association between vaccination status (vaccinated vs unvaccinated) and the risk of being admitted to an ICU for COVID-19 pneumonia was evaluated by analyzing the time course of daily relative risk (RR). For each day, starting from August 1, 2021, the RR was calculated as follows: *RR = (N°ICU_V_ / N°POP_V_) / (N°ICU_UnV_ / N°POP_UnV_),* where *N°ICU_V_* is the number of vaccinated patients admitted to an ICU; *N°POP_V_* is the total number of vaccinated individuals in Lombardy; *N°ICU_UnV_* is the number of unvaccinated patients admitted to an ICU; and *N°POP_UnV_* is the total number of unvaccinated individuals in Lombardy.

The moving average method for time series data was used to understand how the risk of ICU admission for COVID-19 pneumonia changed over time in vaccinated and unvaccinated individuals. For each day, we calculated *N°ICU_V_* as the sum of vaccinated patients admitted to an ICU within the 29-day period from 14 days before and up to 14 days after the selected day. We calculated *N°POP_V_* as the mean number of all vaccinated individuals in Lombardy within the 29-day period from 14 days before and up to 14 days after the selected day. *N°ICU_UnV_* and *N°POP_UnV_* were calculated similarly considering the number of unvaccinated patients admitted to ICU and unvaccinated individuals in Lombardy. In the moving mean calculation, we used as denominator the mean number of individuals in the 29-day window, because we assumed that it is relatively stable because the daily vaccination capacity was limited. The 29-day period was chosen as the best option among several tests (eFigure 2, 3, 4, and 5 in Supplement 1). The time course was then stratified categorizing vaccinated and unvaccinated individuals according to the type of vaccine (mRNA, AdV), time from the administration of the last vaccine dose (dichotomized as more than 120 days, less than or equal to 120 days), sex, age (dichotomized as greater than 65 years of age, less than or equal to 65 years of age), and the combination of time and type of vaccine. See eFigures 6, 7, 8, and 9 in Supplement 1 for additional details.

The daily number of ICU admissions was fitted using a generalized linear regression model with a negative binomial distribution, better suited for over-dispersion of variance. In order to compare the daily risk of ICU admission between vaccinated and unvaccinated populations, the daily size of populations was used as an offset term in the models. Because we were interested in also considering the type of vaccine and the time elapsed since the last vaccine administration, we decided to categorize the variable vaccination status into 4 categories: (1) unvaccinated; (2) AdV vaccine with last administration less than or equal to 120 days; (3) adenovirus vaccine with last administration more than 120 days; mRNA vaccine with last administration ≤120 days; (4) mRNA vaccine with last administration >120 days.

Therefore, a negative binomial regression model^18^ was used to derive incidence rate ratios (IRRs) with 95% CIs comparing the incidence of ICU admission in those vaccinated (according to the type of vaccine and time from vaccination) with those observed in the unvaccinated population. In the multivariable model, age class (less than or equal to 65 years of age; greater than 65 years of age) and sex were considered as confounders.

#### Characteristics and Outcomes of Vaccinated and Unvaccinated Patients Admitted to ICU

No statistical sample size calculation was performed a priori, and the sample size was equal to the number of patients consecutively treated in the participating ICUs during the study period. Categorical variables are reported as frequencies (percentages) and continuous variables as the median and IQR. Categorical variables were compared with χ^2^ test or Fisher exact test, as appropriate. Pairwise comparisons among unvaccinated, mRNA, and AdV were corrected with Bonferroni adjustment.

Continuous variables were compared with Wilcoxon-Mann-Whitney tests (vaccination status) and the Kruskal-Wallis test among unvaccinated, mRNA, and AdV groups with Dwass-Steel-Critchlow-Fligner statistics for multiple comparisons.

#### Multivariable Analysis

We performed multivariable analysis for evaluating ICU mortality and hospital mortality using statistically significant risk factors selected by backward stepwise logistic regression (risk factor–removing criteria: *P* > .05). Both models included patient demographics, vaccination status, number of comorbidities (categorized as 0, 1, 2 and greater than 2), type of comorbidity (hypertension, heart disease, diabetes [type 2], COPD, CKD, liver disease, malignant neoplasms), chronic immunosuppressive therapy, and Pao_2_/FiO_2_ at ICU admission. The odds ratio (OR) and the 95% CI were reported. Hosmer-Lemeshow test was performed to assess the goodness of fit of the models.

Mortality in patients admitted to an ICU for COVID-19 pneumonia cannot consider a rare event, so OR could not be approximated to RR. Therefore, we performed the same multivariable analysis for ICU and hospital mortality using log-binomial regression model in order to have more accurate RR estimates.

No sensitivity analysis was performed. No statistical methods were used to impute missing data.

Two-sided *P* < .05 were considered statistically significant. Data analyses were performed using Python 3.9 with Anaconda 2020.11, SAS Studio 3.8 enterprise edition (SAS Institute) and R version 4.1 (Project for Statistical Computing). IRR were computed from August 1, 2021, to January 31, 2022; ICU and baseline characteristics and outcomes of vaccinated and unvaccinated patients admitted to an ICU were analyzed from August 1 to December 15, 2021.

## Results

The vaccination campaign in Lombardy started on December 27, 2020, when the population was 10 107 674 inhabitants; median (IQR) age was 48 (28-64) years and 5 154 914 (51.0%) were female. On January 31, 2022, 7 863 417 individuals were vaccinated, with a median (IQR) age of 53 (33-68) years and 4 010 343 (51.4%) were female; 6 251 417 (79.5%) received an mRNA vaccine, 550 439 (7.0%) received an AdV vaccine, 1 061 561 (13.5%) received a mix of vaccines and 4 497 875 (57.2%) were boosted (eFigures 6, 7, 8, and 9 in Supplement 1).

From December 27, 2020, to January 31, 2022, 1 657 592 individuals were infected with SARS-CoV-2, approximately 67 000 (data were not available for 9 days) were hospitalized and 5269 were admitted to an ICU for COVID-19 pneumonia without a previous SARS-CoV-2 infection and did not receive a mix of vaccines (Figure 1). The median (IQR) age was 66 (58-73) years, 1581 (30.0%) were female, and ICU mortality was 1868 (35.5%); 4923 ICU patients (93.4%) were unvaccinated, 198 (3.8%) received mRNA vaccines, and 148 (2.8%) received AdV vaccines (eTable 4 and eFigure 10 in Supplement 1).

From December 27, 2020, to July 31, 2021, viral genotyping was available in 417 patients admitted to an ICU, 350 patients (83.9%) were infected with the Alpha variant of COVID-19. From August 1, 2021, to December 15, 2021, viral genotyping was available in 309 ICU patients, and 297 patients (96.1%) were infected with the Delta variant. After December 15, 2021, the prevalence of the Delta variant decreased to 157 ICU patients (68.0%) out of 231 patients with genotyping due to the appearance of Omicron, genotyped in 58 patients (25.1%) (eTable 5, eFigures 11, 12, and 13 in Supplement 1). A total of 19 ICU patients (8.2%) demonstrated more than 1 genotype.

### Association of Vaccination With ICU Admission

**Figure 2.**
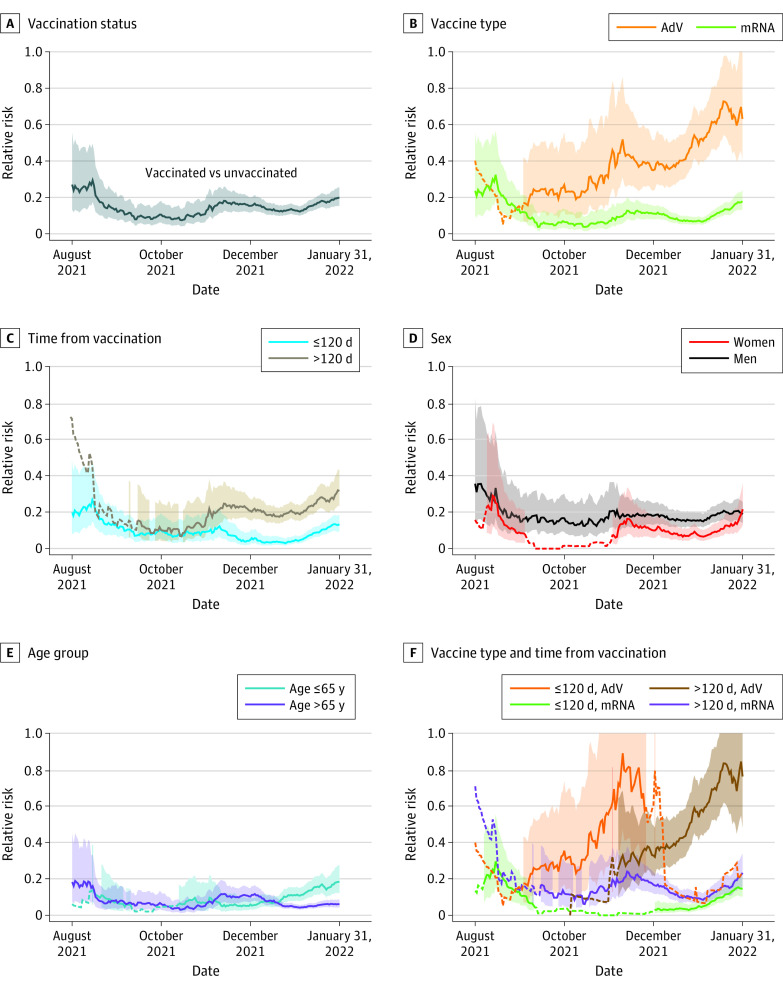
Relative Risk of Intensive Care Unit Admission for COVID-19 Pneumonia Over Time The trend over time of the relative risk of intensive care unit admission for COVID-19 pneumonia stratified by vaccination status (A), by vaccine type (B), by time from vaccination (C), by sex (D), by age (E), and by a combination of vaccine type and time from vaccination (F), considering as reference category the unvaccinated individuals. The dashed lines represent less than 5 vaccinated or unvaccinated patients admitted to an intensive care unit. AdV indicates vaccinated with adenoviral vector vaccines; mRNA, vaccinated with mRNA vaccines.

Time trends of RR showed how vaccination status was associated with the risk of ICU admission for COVID-19 pneumonia during the study period (Figure 2). Vaccination significantly decreased the risk of admission to ICU for COVID-19 pneumonia with an unadjusted IRR of 0.15 (95% CI, 0.13-0.17; *P* < .001) (Figure 2A, eTable 6 in Supplement 1). Adjusting for age, sex, time from the last administered dose and vaccine type, the risk of ICU admission remained lower in vaccinated individuals compared with those unvaccinated (Table 1). We observed the lowest IRR (0.03; 95% CI, 0.03-0.04; *P* < .001) among individuals who had a time from the last administered dose of an mRNA vaccine of less than or equal to 120 days and the highest IRR (0.21; 95% CI, 0.19-0.24; *P* < .001) among individuals who had a time from the last administered dose of an AdV vaccine of more than 120 days (Table 1). Even 4 months after vaccine administration, vaccinated individuals still had a 4.5 times lower risk of ICU admission compared with unvaccinated individuals (eTable 6 in Supplement 1).

**Table 1.** Association of Vaccination With ICU Admission^a^

Multivariable negative binomial model	IRR (95%CI)	*P* value
Vaccination type and time from vaccination		
No vaccine	[Reference]	
AdV ≤120 d	0.06 (0.05-0.07)	<.001
mRNA ≤120 d	0.03 (0.03-0.04)	<.001
AdV >120 d	0.21 (0.19-0.24)	<.001
mRNA >120 d	0.09 (0.08-0.10)	<.001
Age, y		
≤65	0.18 (0.16-0.19)	<.001
>65	[Reference]	
Sex		
Female	0.31 (0.28-0.33)	<.001
Male	[Reference]	

Abbreviations: AdV, adenoviral vector vaccine; ICU, intensive care unit; IRR, incidence rate ratio.

^a^ Results of multivariable generalized linear model with a negative binomial distribution and a log offset of population to account for different observations over time. Association was estimated using the IRR and its related 95% CI.

Moreover, after adjusting for confounders, female individuals had a lower risk of ICU admission for COVID-19 pneumonia compared with male individuals (IRR, 0.31; 95% CI, 0.28-0.33; *P* < .001), as well as younger people (less than or equal to 65 years of age) compared with older (greater than 65 years of age) with an IRR of 0.18 (95% CI, 0.16-0.19; *P* < .001). All IRR estimates by univariable and multivariable negative binomial regression models are reported in eTable 6 in Supplement 1.

### Characteristics and Outcomes of Vaccinated and Unvaccinated Patients Admitted to an ICU

From August 1, 2021, to December 15, 2021, on average, 6 847 146 (67%) individuals have been vaccinated while 3 302 631 (33%) individuals were not vaccinated. During this period, 553 patients without a previous SARS-CoV-2 infection and who did not receive a mix of vaccines were admitted to one of the 34 ICUs of the Lombardy COVID-19 ICU Network for acute respiratory failure due to COVID-19 pneumonia (Table 2). Prior to ICU admission, 414 patients (74.9%) were not vaccinated, and 139 (25.1%) were vaccinated (81 [14.7%] with mRNA vaccines [75 (54.0%) BNT162b2; 6 (4.3%) mRNA-1273] and 58 (10.5%) with AdV vaccines [51 (36.7%) ChADOx1-S; 7 (5.0%) Ad26.COV2]). Only 2 patients admitted to ICU had received a booster dose. All baseline characteristics, clinical parameters, and outcomes are reported in Table 2, also stratifying them by vaccination status and type of vaccine.

**Table 2.** Baseline Characteristics and Outcomes^a^

Characteristics	All, No. (%)	Unvaccinated, No. (%)	Vaccinated, No. (%)	*P* value for unvaccinated vs vaccinated	mRNA vaccines, No. (%)	AdV vaccines, No. (%)	*P* value			
							Type of vaccine^b^	Unvaccinated vs AdV	Unvaccinated vs mRNA	AdV vs mRNA
No.	553 (100)	414 (74.9)	139 (25.1)		81 (14.6)	58 (10.5)				
Age, median (IQR), y	64 (54-72)	60 (51-69)	72 (66-76)	<.001	71 (66-76)	72.5 (67-77)	<.001	<.001	<.001	.87
No.	552	413	139		81	58				
Female	191 (34.5)	162 (39.1)	29 (20.9)	<.001	20 (24.7)	9 (15.5)	<.001	.001	.04	.57
Comorbidities										
No. of comorbidities, median (IQR)	1 (0-2)	0 (0-1)	2 (1-3)	<.001	2 (1-4)	1 (1-3)	<.001	<.001	<.001	.01
Hypertension	204 (36.9)	116 (28.0)	88 (63.3)	<.001	52 (64.2)	36 (62.1)	<.001	<.001	<.001	.99
Hypercholesterolemia	76 (13.7)	32 (7.7)	44 (31.7)	<.001	27 (33.3)	17 (29.3)	<.001	<.001	<.001	.99
Heart disease	75 (13.6)	35 (8.5)	40 (28.8)	<.001	27 (33.3)	13 (22.4)	<.001	.003	<.001	.48
Diabetes (type 2)	85 (15.4)	47 (11.4)	38 (27.4)	<.001	28 (34.6)	10 (17.2)	<.001	.59	<.001	.07
COPD	12 (2.2)	7 (1.7)	5 (3.6)	.18	2 (2.5)	3 (5.2)	.17	NA	NA	NA
CKD	17 (3.1)	6 (1.5)	11 (7.9)	<.001	8 (9.9)	3 (5.2)	<.001	.26	.002	.99
Liver disease	12 (2.2)	8 (1.9)	4 (2.9)	.51	4 (4.9)	0 (0.00)	.12	NA	NA	NA
Malignant neoplasm	54 (9.8)	24 (5.8)	30 (21.6)	<.001	22 (27.2)	8 (13.8)	<.001	.07	<.001	.18
Other	100 (18.1)	54 (13.0)	46 (33.1)	<.001	36 (44.4)	10 (17.2)	<.001	.99	<.001	.002
No comorbidities	260 (47.0)	238 (57.5)	22 (15.8)	<.001	10 (12.4)	12 (20.7)	<.001	<.001	<.001	.55
Immunodepression										
Solid organ transplantation	10 (1.8)	5 (1.2)	5 (3.6)	.07	5 (6.2)	0 (0.00)	.01	.99	.04	.23
Autoimmune disease	23 (4.2)	12 (2.9)	11 (7.9)	.01	10 (12.4)	1 (1.7)	.002	.99	.003	.08
Chronic immunosuppressor treatment	185 (33.5)	125 (30.2)	60 (43.2)	.005	41 (50.6)	19 (32.8)	.002	.99	.001	.11
Disease chronology										
Time from last dose to ICU, median (IQR), d	NA	NA	144 (108-183)	NA	164 (128-199)	131.5 (96-147)	<.001	NA	NA	NA
Time from hospital admission to ICU admission, median (IQR), d	2 (0-5)	2 (0-4)	2 (0-6)	.40	2 (0-6)	3 (1-5)	.27	NA	NA	NA
No.	498	363	135		78	57				
Time from diagnosis to ICU admission, median (IQR), d	5 (1-8)	5 (1-9)	4 (1-7)	.13	4 (1-7)	5 (2-8)	.23	NA	NA	NA
No.	506	367	139		81	58				
Respiratory support at ICU admission										
Invasive ventilation	320 (60.2)	241 (60.6)	79 (50.0)	.74	40 (52.0)	39 (68.4)	.15	NA	NA	NA
No.	532	398	134		77	57				
NIV	67 (12.6)	49 (12.3)	18 (13.4)	.72	10 (13.0)	8 (14.0)	.92	NA	NA	NA
No.	534	400	134		77	57				
CPAP	115 (21.5)	90 (22.5)	25 (18.7)	.35	18 (23.4)	7 (12.3)	.20	NA	NA	NA
No.	534	400	134		77	57				
ECMO	1 (0.2)	0 (0.0)	1 (0.9)	.23	0 (0.0)	1 (2.1)	.01	NA	.23	.87
No.	464	356	108		61	47				
Prone Position	110 (23.1)	92 (25.4)	18 (15.8)	.03	6 (9.5)	12 (23.5)	.02	.02	.99	.21
No.	476	362	114		63	51				
Respiratory parameters										
Pao_2_, median (IQR), mm Hg	83 (70-100)	82 (70-98)	84 (73-109)	.16	87 (71-120)	83 (76-98)	.33	NA	NA	NA
No.	455	351	104		59	45				
FiO_2_, median (IQR), %	70 (60-90)	70 (60-90)	60 (52.5-80)	.01	60 (60-80)	60 (50-80)	.04	.11	.17	.88
No.	470	358	112		63	49				
Pao_2_/FiO_2_, median (IQR), mm Hg	124 (93-163)	120 (90-158)	138 (100-180)	.007	142.5 (94-183)	137 (114-170)	.02	.11	.09	.95
No.	454	351	103		58	45				
PEEP, median (IQR), cmH_2_O	10 (8-12)	10 (8-12)	10 (10-12)	.47	10 (8-12)	10 (10-12)	.77	NA	NA	NA
No.	456	347	109		63	46				
Outcomes										
ICU mortality	152 (27.5)	107 (25.9)	45 (32.4)	.14	28 (34.6)	17 (29.3)	.26	NA	NA	NA
ICU length of stay, median (IQR), d	13 (7-24)	13 (7-24)	13 (7-25)	.90	12 (6-25)	14.5 (8-23)	.91	NA	NA	NA
Hospital mortality	167 (32.6)	112 (29.9)	55 (40.2)	.03	37 (46.3)	18 (31.6)	.02	.99	.01	.25
No.	512	375	137		80	57				
Length of hospital stay, median (IQR), d	25 (17-36)	25 (17-36)	25 (17-37)	.91	25 (15-39)	25 (19-33)	.97	NA	NA	NA
No.	498	363	135		78	57				
Mechanical Ventilation during ICU stay, median (IQR), d	9 (3-21)	9 (3-21)	10 (3-23)	.44	9 (1-23)	11.5 (6-24)	.49	NA	NA	NA
No.	545	408	137		79	58				
Invasive ventilation ICU	438 (80.4)	324 (79.4)	114 (83.2)	.33	62 (78.5)	52 (89.7)	.17	NA	NA	NA
No.	545	408	137		79	58				
ECMO	22 (4.2)	20 (5.1)	2 (1.6)	.13	1 (1.4)	1 (1.9)	.33	NA	NA	NA
No.	522	396	126		73	53				
Prone position	394 (74.3)	317 (79.3)	77 (59.2)	<.001	41 (54.7)	36 (65.5)	<.001	.06	<.001	.65
No.	530	400	130		75	55				

Abbreviations: CKD, chronic kidney disease; COPD, chronic obstructive pulmonary disease; CPAP, continuous positive airway pressure; ECMO, extracorporeal membrane oxygenation; FiO_2_, fraction of inspiratory oxygen; ICU, intensive care unit; NA, not applicable; NIV, noninvasive ventilation; Pao_2_, arterial partial pressure of oxygen; PEEP, positive end-expiratory pressure.

^a^ Baseline characteristics and outcomes in vaccinated and unvaccinated patients admitted to ICU from August 1, 2021, to December 15, 2021. eTable 8 in Supplement 1 presents number of missing and available data.

^b^ Comparison among the 3 groups of patients: unvaccinated, vaccinated with mRNA, and vaccinated with AdV.

In detail, compared with unvaccinated patients admitted to an ICU, vaccinated patients were older (median [IQR] age was 72 [66-76] years vs 60 [51-69] years; *P* < .001) and had more comorbidities: prevalence of patients with at least 1 comorbidity was 84.2% (117 vaccinated patients) vs 42.5% (176 unvaccinated patients) (*P* < .001) and the median (IQR) number of comorbidities was 2 (1-3) vs 0 (0-1) (*P* < .001). Among vaccinated patients, the mRNA group had more comorbidities compared with the AdV group (*P* = .01). The median (IQR) time between COVID-19 diagnosis and ICU admission was 5 (1-8) days and no statistical differences were detected according to vaccination status and to the type of vaccine. Compared with vaccinated, unvaccinated participants had higher median (IQR) FiO_2_ (60.0% [52.5%-80.0%] vs 70.0% [60.0%-90.0%]; *P* = .01) and had lower median (IQR) Pao_2_/FiO_2_ (138 [100-180] mm Hg vs 120 [90-158] mm Hg; *P* = .007), whereas PEEP was similar with a median (IQR) of 10 (8-12) cmH_2_O. During an ICU stay, 80.4% (438 out of 545 patients) underwent invasive mechanical ventilation, prone positioning was performed in 74.3% (394 of 530 patients) (59.2% in vaccinated patients [77 of 130] and 79.3% in unvaccinated [317 of 400]; *P* < .001) and 4.2% (22 of 522) required ECMO.

The median (IQR) length of stays were 13 (7-24) days for an ICU and 25 (17-36) days for a hospital, and were similar in vaccinated and unvaccinated patients. ICU mortality was similar between unvaccinated and vaccinated patients (25.9% [n = 107] vs 32.4% [n = 45]; *P* = .14), while hospital mortality was higher in vaccinated patients (29.9% [112 of 375 ] vs 40.2% [55 of 137], *P* = .03).

### Multivariable Analysis

In the multivariable regression analysis, increased ICU mortality was associated with older age, female status, premorbid heart disease and lower Pao_2_/FiO_2_ values. Increased hospital mortality was associated with older age, premorbid heart disease and lower Pao_2_/FiO_2_ values (Table 3; eTable 7 in Supplement 1). In both analyses, vaccination status was not detected as a statically significant factor for mortality.

**Table 3.** Multivariable Analyses^a^

Outcome	Category	Multivariable log-binomial model	
		RR (95% CI)	*P* value
Outcome: ICU mortality^b^			
Age, y	5-y increments	1.23 (1.15-1.33)	<.001
Sex	Female vs male	1.42 (1.07-1.89)	.02
Heart disease	Yes vs no	1.62 (1.21-2.18)	.001
Pao_2_/FiO_2_, mm Hg	20 mm Hg increments	0.88 (0.84-0.93)	<.001
Outcome: Hospital mortality^c^			
Age, y	5-y increments	1.24(1.18-1.32)	<.001
Heart disease	Yes vs no	1.40 (1.08-1.82)	.02
Pao_2_/FiO_2_, mm Hg	20 mm Hg increments	0.93 (0.89-0.98)	.005

Abbreviations: FiO_2_, fraction of inspiratory oxygen; ICU, intensive care unit; Pao_2_, arterial partial pressure of oxygen; RR, relative risk.

^a^ Risk factors were defined using a backward stepwise approach. The list of possible risk factors included: age, sex, vaccination status, type of comorbidity (hypertension, heart disease, diabetes, chronic kidney disease, liver disease, chronic obstructive pulmonary disease, malignant neoplasm), immunosuppressive therapy, number of comorbidities (categorized as 0, 1, 2, and >2), Pao_2_/FiO_2_ at ICU admission.

^b^ Multivariable models on ICU mortality were carried out on 454 individuals.

^c^ Multivariable models on hospital mortality were carried out on 454 individuals.

## Discussion

In this cohort study, we found that vaccination with COVID-19 vaccines based on mRNA technology or adenovirus vectors was associated with a reduced risk of being admitted to the ICU. Vaccinated patients admitted to the ICU for COVID-19 pneumonia were older and had more comorbidities compared with unvaccinated patients. Adjusting for confounders, ICU and hospital mortality rates were not different between vaccinated and unvaccinated patients.

Our analyses, similar to previously published data,^19,20,21^ show that the risk of ICU admission for COVID-19 pneumonia in vaccinated individuals was 15% of the risk of unvaccinated individuals, meaning that being unvaccinated resulted in almost 7-times higher risk of being admitted to an ICU. When adjusted for age and sex, the risk was 14 times higher. Indeed, most of the prior research evaluated the role of vaccination in decreasing the risk of developing severe disease without specifically investigating admission to ICU and showed an efficacy ranging from 75% to 96%.^1,2,3^

Our results, without considering the vaccination status, found that male individuals had a 2-times higher risk of being admitted to an ICU compared with female individuals, and that individuals older than 65 years had a 3-times higher risk of ICU admission compared with younger individuals.

All vaccines administered during the vaccination campaign in Lombardy were associated with decreased risk of developing a severe COVID-19 pneumonia leading to ICU admission, but mRNA vaccines showed more than 2 times lower risk than AdV vaccines, after adjusting for age and sex. Other studies showed an increased risk of developing severe COVID-19 among vaccinated individuals of advanced age, especially those aged at least 80 years.^22,23,24,25^ Cerqueira-Silva et al^5^ compared ChADOx1-S (vaccine using adenovirus vector) to Sinovac-CoronaVac (inactivated virus), and reported an efficacy in decreasing the risk of ICU admission up to 91% with ChADOx1-S (vaccine using adenoviral vector) and 90% with Sinovac-CoronaVac (inactivated virus)^5^ whereas Dickerman et al^4^ showed higher efficacy of mRNA-1273 in preventing ICU admission compared with BNT162b2. We did not find data in the literature comparing the effectiveness of mRNA and AdV vaccines. Considering time from the last administrated vaccine dose, we detected a positive association between time and risk of ICU admission, as reported in the literature.^6,21,22,23,24,26,27,28,29,30^

Even 4 months after vaccine administration, vaccinated individuals still had a 4.5-times lower risk of ICU admission compared with unvaccinated individuals. Furthermore, mRNA vaccines maintained a greater and more prolonged effectiveness in preventing ICU admission compared with AdV.

During the vaccination campaign, Lombardy experienced 2 pandemic waves. The first wave (early 2021) was characterized by Alpha variant predominance and a low prevalence of vaccinated citizens (less than 1‰ [parts per thousand] of patients admitted to ICU were vaccinated). During the summer of 2021, the Delta variant appeared and quickly became predominant while about 50% of the Lombardy population had been fully vaccinated. Consequently, the percentage of vaccinated patients admitted to ICU progressively increased, ranging from 30% to 40%. The demographics and baseline health status of ICU patients with COVID-19 pneumonia varied dramatically according to the vaccination status. Unvaccinated patients were 12 years younger than vaccinated patients, had fewer comorbidities and a lower prevalence of immunosuppressive therapy at baseline but showed a more severe acute respiratory failure at ICU admission. Among vaccinated patients, the mRNA group had more comorbidities, likely due to the criteria for vaccination eligibility specified by the Italian campaign (eTable 1 in Supplement 1).

Despite these important differences, we did not observe a substantial difference in ICU and hospital length of stay and in ICU mortality between vaccinated and unvaccinated patients. At variance, hospital mortality was higher in the mRNA vaccine group, possibly due to the older age and higher number of comorbidities. Indeed, accounting for all statistically significant confounders, we did not find an association between hospital mortality and vaccination status.

Available data describing ICU mortality is very heterogenous due to differences in patient demographics, baseline health status, and need for respiratory support. Indeed, the reported crude mortality ranged from 23% to 33% for vaccinated patients and 22% and 29% for unvaccinated patients.^15,16^

In Italy, because of safety concerns, a substantial number of individuals were vaccinated with AdV-based vaccines and then boosted with an mRNA vaccine (in Lombardy, 822 507 after ChAdOx1-S, and 105 829 after Ad26.COV2) (eFigure 14 in Supplement 1). Evidence suggests that heterologous vaccination regimens mixing AdV and mRNA vaccines elicits an enhanced quality of the B- and T-cell responses.^31^ Unfortunately, the low number of individuals available in this cohort prevented the assessment of the impact of heterologous vaccination on ICU admission and outcome. Given the broad usage worldwide of mixed heterologous vaccination regiments^32^ it will be important to assess their association with ICU admission and outcome.

### Limitations

This study has several limitations. First, it is a retrospective cohort study of data extracted from different data sets of the Lombardy Regional Health Service. Second, because COVID-19 vaccines were not indicated for individuals with recent SARS-CoV-2 infection, the prevalence of previous infections was probably higher in unvaccinated individuals. Thus, accounting for previous infections may lead to the computation of a slightly lower risk of ICU admission. Third, we focused on the SARS-CoV-2 Delta variant infection; we cannot exclude that other variants could have a different association between vaccination and mortality. Fourth, the data set used for the analysis did not provide clinically relevant information such as superinfections, medical and pharmaceutical interventions, or the cause of death. Fifth, individuals vaccinated with a combination of mRNA and AdV technology vaccines were excluded from the analyses due to the low number. Additionally, the known limits of the backward stepwise approach applied to identify factors associated with mortality may have affected our findings.

## Conclusions

This cohort study found that both mRNA and AdV vaccines were associated with a significant reduction of the risk of developing COVID-19–related severe acute respiratory failure requiring ICU admission. Female sex, younger age, vaccination, and a shorter time from last vaccine dose administration were all independently associated with reduced ICU admission risk. Among patients admitted to an ICU for COVID-19 pneumonia, vaccinated patients were older and had more comorbidities compared with unvaccinated patients. Adjusting for age and comorbidities, ICU and hospital mortality were similar between vaccinated and unvaccinated patients.

## Notes

Supplement 1. **eFigure 1.** Vaccination Campaign in Lombardy **eTable 1.** Anamnestic Criteria for Selecting the Type of Vaccine to be Administered, According to the Indication of the Italian Ministry of Health **eTable 2.** Definition of Comorbidities According to the Chronic Diseases Collected in the Regional Databases **eTable 3.** Definition of Immunodepression **eFigure 2.** Relative Risks Without Any Window Function **eFigure 3.** Relative Risks With Window Size of 15 Days **eFigure 4.** Relative Risks with Window Size of 29 Days **eFigure 5.** Relative Risks with Window Size of 43 Days **eFigure 6.** Vaccination Rate Stratified by Type of Vaccine in Lombardy **eFigure 7.** Vaccination Rate Stratified by Booster Dose and Type of Vaccine in Lombardy **eFigure 8.** Vaccination Rate by Age and Type of Vaccine in Lombardy **eFigure 9.** Vaccination Rate by Sex and Type of Vaccine in Lombardy **eTable 4.** Patients Admitted to ICU, December 27, 2021 – January 31, 2022 **eTable 5.** Genotypes Frequency in ICU **eFigure 10.** Number of ICU Admissions According to Vaccination Status **eFigure 11.** Prevalence of SARS-CoV-2 Variants **eFigure 12.** Number of ICU Admissions According to SARS-CoV-2 Variants **eFigure 13.** Prevalence of SARS-CoV-2 Variants in ICU **eTable 6.** Role of Vaccination in Preventing ICU Admission **eTable 7.** Multivariable Analysis - Comparison Between OR and RR Estimated by Multivariable Logistic and Log-Binomial Model, Respectively **eFigure 14.** Relative Risk According to the Different Types of Vaccines **eTable 8.** Number of Missing and Available Data Related to Characteristics in Table 2

Supplement 2. **Nonauthor Collaborators.** COVID-19 Lombardy ICU Network
