# Supplementary material for: Association of COVID-19 Vaccinations With Intensive Care Unit Admissions and Outcome of Critically Ill Patients With COVID-19 Pneumonia in Lombardy, Italy
Source: JAMA Netw Open. 2022 Oct 27;5(10):e2238871. doi: 10.1001/jamanetworkopen.2022.38871 (PMC9614574; doi:10.1001/jamanetworkopen.2022.38871)
Supplement: Supplement 1. — eFigure 1. Vaccination Campaign in Lombardy eTable 1. Anamnestic Criteria for Selecting the Type of Vaccine to be Administered, According to the Indication of the Italian Ministry of Health eTable 2. Definition of Comorbidities According to the Chronic Diseases Collected in the Regional Databases eTable 3. Definition of Immunodepression eFigure 2. Relative Risks Without Any Window Function eFigure 3. Relative Risks With Window Size of 15 Days eFigure 4. Relative Risks with Window Size of 29 Days eFigure 5. Relative Risks with Window Size of 43 Days eFigure 6. Vaccination Rate Stratified by Type of Vaccine in Lombardy eFigure 7. Vaccination Rate Stratified by Booster Dose and Type of Vaccine in Lombardy eFigure 8. Vaccination Rate by Age and Type of Vaccine in Lombardy eFigure 9. Vaccination Rate by Sex and Type of Vaccine in Lombardy eTable 4. Patients Admitted to ICU, December 27, 2021 – January 31, 2022 eTable 5. Genotypes Frequency in ICU eFigure 10. Number of ICU Admissions According to Vaccination Status eFigure 11. Prevalence of SARS-CoV-2 Variants eFigure 12. Number of ICU Admissions According to SARS-CoV-2 Variants eFigure 13. Prevalence of SARS-CoV-2 Variants in ICU eTable 6. Role of Vaccination in Preventing ICU Admission eTable 7. Multivariable Analysis - Comparison Between OR and RR Estimated by Multivariable Logistic and Log-Binomial Model, Respectively eFigure 14. Relative Risk According to the Different Types of Vaccines eTable 8. Number of Missing and Available Data Related to Characteristics in Table 2 [file jamanetwopen-e2238871-s001.pdf]

Supplemental Online Content

Grasselli G, Zanella A, Carlesso E, et al; COVID-19 Lombardy ICU Network. Association of COVID-19 vaccinations with intensive care unit admissions and outcome of critically ill patients with COVID-19 pneumonia in Lombardy, Italy. *JAMA Netw Open.* 2022;5(10):e2238871. doi:10.1001/jamanetworkopen.2022.38871

eFigure 1. Vaccination Campaign in Lombardy

eTable 1. Anamnestic Criteria for Selecting the Type of Vaccine to be Administered, According to the Indication of the Italian Ministry of Health

eTable 2. Definition of Comorbidities According to the Chronic Diseases Collected in the Regional Databases

eTable 3. Definition of Immunodepression

eFigure 2. Relative Risks Without any Window Function

eFigure 3. Relative Risks With Window Size of 15 Days

eFigure 4. Relative Risks with Window Size of 29 Days

eFigure 5. Relative Risks with Window Size of 43 Days

eFigure 6. Vaccination Rate Stratified by Type of Vaccine in Lombardy

eFigure 7. Vaccination Rate Stratified by Booster Dose and Type of Vaccine in Lombardy

eFigure 8. Vaccination Rate by Age and Type of Vaccine in Lombardy

eFigure 9. Vaccination Rate by Sex and Type of Vaccine in Lombardy

eTable 4. Patients Admitted to ICU, December 27, 2021 – January 31, 2022

eTable 5. Genotypes Frequency in ICU

eFigure 10. Number of ICU Admissions According to Vaccination Status

eFigure 11. Prevalence of SARS-CoV-2 Variants

eFigure 12. Number of ICU Admissions According to SARS-CoV-2 Variants

eFigure 13. Prevalence of SARS-CoV-2 Variants in ICU

eTable 6. Role of Vaccination in Preventing ICU Admission

eTable 7. Multivariable Analysis - Comparison Between OR and RR Estimated by Multivariable Logistic and Log-Binomial Model, Respectively

eFigure 14. Relative Risk According to the Different Types of Vaccines

eTable 8. Number of Missing and Available Data Related to Characteristics in Table 2

This supplemental material has been provided by the authors to give readers additional information about their work.

© 2022 Grasselli G et al. *JAMA Network Open.*

Additional Introduction

eFigure 1 - Vaccination campaign in Lombardy.

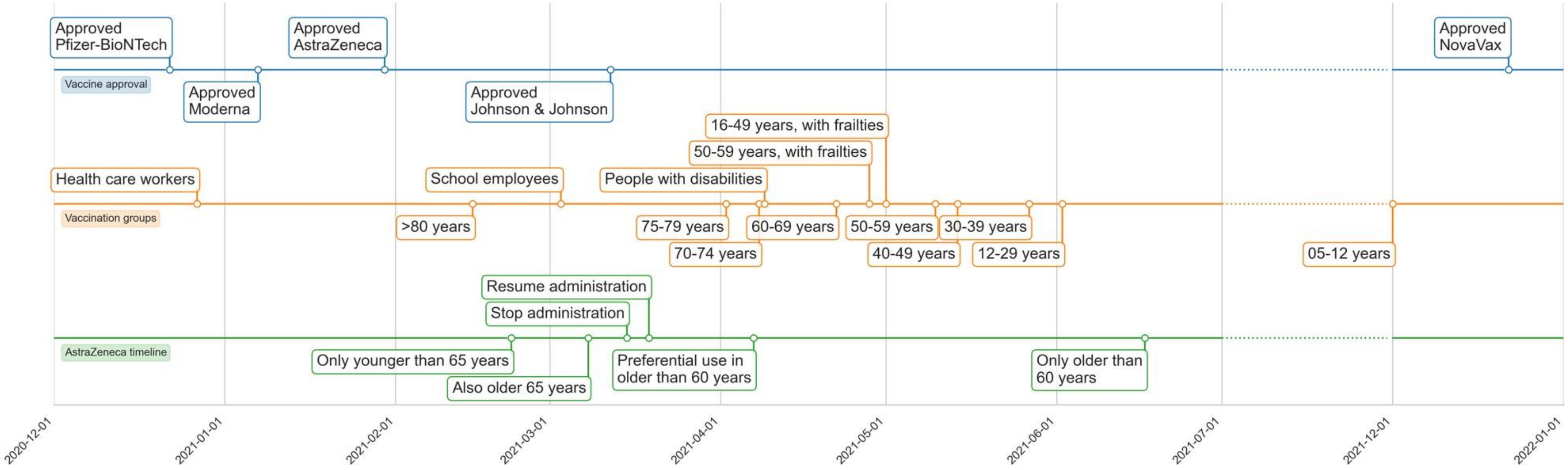

eFigure 1 shows the timeline of the vaccination campaign in Lombardy. The scheme is divided into three parts indicating: vaccine approval (blue line), target population (vaccination group, orange line), AstraZeneca administration rules over time (green line). Abbreviations: Pfizer-BioNTech, Comirnaty BNT162b2, Pfizer-BioNTech; Moderna, Spikevax mRNA-1273, Moderna; AstraZeneca, Vaxzevria ChADOx1-S, AstraZeneca; Johnson & Johnson, Ad26.COV2 Janssen, Johnson & Johnson; NovaVax, Nuvaxovid NVX-CoV2373, Novavax.

© 2022 Grasselli G et al. *JAMA Network Open*.

eTable 1. Anamnestic criteria for selecting the type of vaccine to be administered, according to the indication of the Italian Ministry of Health.

Patients with the following comorbidities preferentially received mRNA vaccines:
- Idiopathic pulmonary fibrosis;
- All other respiratory conditions requiring oxygen supplementation;
- Heart failure (III- IV NYHA);
- Cardiogenic shock;
- Amyotrophic lateral sclerosis;
- Multiple sclerosis;
- Muscular dystrophy;
- Cerebral palsy;
- Immunodeficient patients, or patients on therapies with immunosuppressor drug;
- Myasthenia gravis;
- Dyssimune neurological disorders;
- Type 1 diabetes mellitus ;
- Type 2 diabetes mellitus requiring at least two different medications, or with at least two complications;
- Addison disease;
- Panhypopituitarism;
- Chronic dialysis;
- All severe pulmonary diseases/pulmonary function impairment or severe immunodeficiency;
- Cirrhosis;
- Cerebral ischemic-hemorrhagic event with neurological or cognitive impairment;
- Stroke in 2020 with a ranking score ≥ 3;
- Malignancy in severe stage, not remission;
- Immunosuppressive drugs for malignancy in the last six months;
- Thalassemia, sickle cell anemia;
- Down syndrome;
- All patients in the waiting list for solid organ transplantation;
- All patients in the waiting list or underwent (after three months and before twelve months) a hematopoietic stem cell donation;
- All patients underwent a hematopoietic stem cell donation, even after twelve months, with chronic graft versus host disease and immunosuppressive treatment;
- BMI > 35;
- AIDS or < 200 CD4.

© 2022 Grasselli G et al. *JAMA Network Open.*

Additional Methods

eTable 2. Definition of comorbidities according to the chronic diseases collected in the Regional databases.

**Chronic diseases**	**Hypertension**	**Hypercholesterolemia**	**Diabetes mellitus type 2**	**Heart disease**	**Malignancy**	**COPD**	**Chronic kidney disease**	**Liver disease**	**Other**
Arterial hypertension	■								
Hypercholesterolemia, familiar and not		■							
Diabetes mellitus type 2			■						
Ischemic cardiomyopathy				■					
Non arrhythmic cardiomyopathy				■					
Arrhythmic cardiomyopathy				■					
Hypothyroidism									■
Neoplasia, follow-up					■				
Neoplasia, remission					■				
Active neoplasia					■				
Heart failure				■					
COPD						■			
Chronic kidney disease							■		
Chronic hepatitis								■	
Asthma									■
Cerebral vasculopathy									■
Arterial vasculopathy									■
Valvular cardiomyopathy				■					
Diabetes mellitus type 2, complicated			■						
Hashimoto thyroiditis									■
Venous vasculopathy									■
Epilepsy									■
Rheumatoid arthritis									■
Basedow-Graves disease and hyperthyroidism									■
Psoriasis and psoriatic arthritis									■
Inactive transplant									■
Hepatic cirrhosis								■	
Crohn disease and ulcerative colitis									■
HIV positive and overt aids									■
Parkinson e Parkinsonisms									■
Respiratory failure/ oxygen therapy									■

© 2022 Grasselli G et al. *JAMA Network Open.*

Chronic diseases	Hypertension	Hypercholesterolemia	Diabetes mellitus type 2	Heart disease	Malignancy	COPD	Chronic kidney disease	Liver disease	Other
Systemic lupus erythematous									■
Nervous system and sensory organs diseases									■
Myasthenia gravis									■
Hyper and hypoparathyroidism									■
Osteomuscular and connective tissue diseases									■
Endocrine, nutritional and metabolic diseases									■
Multiple sclerosis									■
Diabetes mellitus type 1, complicated									■
Diabetes mellitus type 1									■
Cardiovascular diseases									■
Congenital malformations									■
Chronic pancreatitis									■
Autoimmune haemolytic anaemias									■
Dialysis							■		
Gastrointestinal diseases									■
Systemic sclerosis									■
Ankylosing spondylitis									■
Active transplant									■
Skin and hypodermic tissue disease									■
Cushing syndrome									■
Dementia									■
Sjogren disease									■
Hematic and hematopoietic organs disease									■
Addison syndrome									■
Tumors					■				

Abbreviations. COPD, chronic obstructive pulmonary disease.

eTable 3. Definition of immunodepression.

Variable	Definition
Immunodepression	Presence of at least one among:<br>▪ solid organ transplantation<br>▪ autoimmune disease<br>▪ chronic immunosuppressor therapy
Autoimmune disease	Presence of at least one among:<br>▪ diabetes type I<br>▪ rheumatoid arthritis<br>▪ systemic lupus erythematosus<br>▪ Sjogren disease<br>▪ connective tissue diseases<br>▪ myasthenia gravis<br>▪ Addison disease<br>▪ Basedow disease and hyperthyroidism<br>▪ systemic sclerosis<br>▪ anchylosing spondylitis<br>▪ Hashimoto thyroiditis
Chronic immunosuppressor therapy	List of drugs: Imiquimod, Ingenol mebutate, Fludrocortisone, Betamethasone, Dexamethasone, Methylprednisolone, Prednisolone, Prednisone, Triamcinolone, Hydrocortisone, Cortisone, Cyclophosphamide, Melphalan, Bendamustine, Methotrexate, Fludarabine, Cytarabine, Flurouracil, Capecitabine, Azacitidine, Fluorouracil, associations, Vincristin, Etoposide, Doxorubicin, Mitomycin, Imatinib, Erlotinib, Osimertinib, Alectinib, Ruxolitinib, Ibrutinib, Nintedanib, Gilteritinib, Carboplatin, Oxaliplatin, Rituximab, Trastuxumab, Bavacixumab, Brentuximab vedotin, Pertuxumab, Obintuxumab, Nivolumab, Pembrolizumab, Daratumumab, Dasatinib, Ibrutinib, Nintedanib, Palbociclib, Bortezomib, Carfilzomib, Hydroxycarbamide, Bortexomib, Carfilzomib, Idelalisib, Venetoclax, Leuprorelin, Triptorelin, Tamoxifen, Fuluestrant, Bicalutramide, Enzalutamide, Anastrozole, Letrozole, Exemestane, Degarelix, Abiraterone, Filgrastim, Lenogastim, Pegfilgastim, Lipefilgrastim, Interferon alfa 2°, Glatiramer acetate, Mycophenoleic acid, Sirolimus, Leflunomide, Everolimus, Natalizumab, Abatacept, Fingolimod, Tofacitinib, Ocrelizumab, Baricitinib, Etanercept, Infliximab, Adalimumab, Golimumab, Anakirna, Tocilizumab, Secukinumab, Brodalumab, Serilumab, Risankuzumab, Cyclosporine, Tacrolimus, Azathioprine, Thalidomide, Methotrexate, Lenalidomide, Pirfenidone, Pomalidomide, Dimetilfumarato, Other therapeutics radio-drugs

Time trend analysis of the risk of ICU admission for COVID-19 pneumonia.

The association between vaccination status (vaccinated *versus* unvaccinated) and the risk of being admitted to the ICU for COVID-19 pneumonia was evaluated by analyzing the time course of daily relative risk (RR). For each day, starting from August 1, 2021, the RR was calculated as follows:

$$RR = \frac{\frac{N°ICU_V}{N°POP_V}}{\frac{N°ICU_{UnV}}{N°POP_{UnV}}}$$

where:

- $N°ICU_V$: number of vaccinated individuals admitted to the ICU
- $N°POP_V$: total number of vaccinated individuals in Lombardy
- $N°ICU_{UnV}$: number of unvaccinated individuals admitted to the ICU
- $N°POP_{UnV}$: total number of unvaccinated individuals in Lombardy

The moving average method for time series data was used to understand how the risk of ICU admission for COVID-19 pneumonia changed over time in vaccinated and unvaccinated individuals. For each day, we considered the number of vaccinated and unvaccinated individuals admitted to the ICU as the sum of vaccinated and unvaccinated patients admitted to the ICU within the 29-day period from 14 days before and up to 14 days after the selected day. We considered the total number of vaccinated and unvaccinated individuals in Lombardy as the average of the number of all vaccinated and unvaccinated individuals in Lombardy within the 29-day period from 14 days before and up to 14 days after the selected day. In the moving average calculation, we used as denominator the average number of individuals in the 29-days window, because we assumed that it is relatively stable since the daily vaccination capacity was limited. The 29-day period was chosen from several window sizes. We report here tests performed on 1 day (without any windowing function, Figure S2), 15 days (Figure S3), 29 days (Figure S4) and 43 days (Figure S5).

As an example, the RR of being admitted to the ICU for COVID-19 pneumonia on October 1st, 2021, was computed as:

- $N°ICU_V$: number of vaccinated patients admitted to the ICU between September 17, 2021 to October 15, 2021, (16 patients)
- $N°POP_V$: average number of all the vaccinated individuals in Lombardy between September 17, 2021 to October 15, 2021 (6967394 individuals)
- $N°ICU_{UnV}$: number of unvaccinated patients admitted to the ICU between September 17, 2021 to October 15, 2021 (70 patients)
- $N°POP_{UnV}$: average number of all the unvaccinated individuals in Lombardy between September 17, 2021 to October 15, 2021 (3182436 individuals)

$$RR, V\ vs\ UnV, \text{October } 1, 2021 = \frac{\frac{16}{6967394}}{\frac{70}{3182436}} = 0.10$$

© 2022 Grasselli G et al. *JAMA Network Open.*

Additional Results

eFigure 2. Relative risks without any window function.

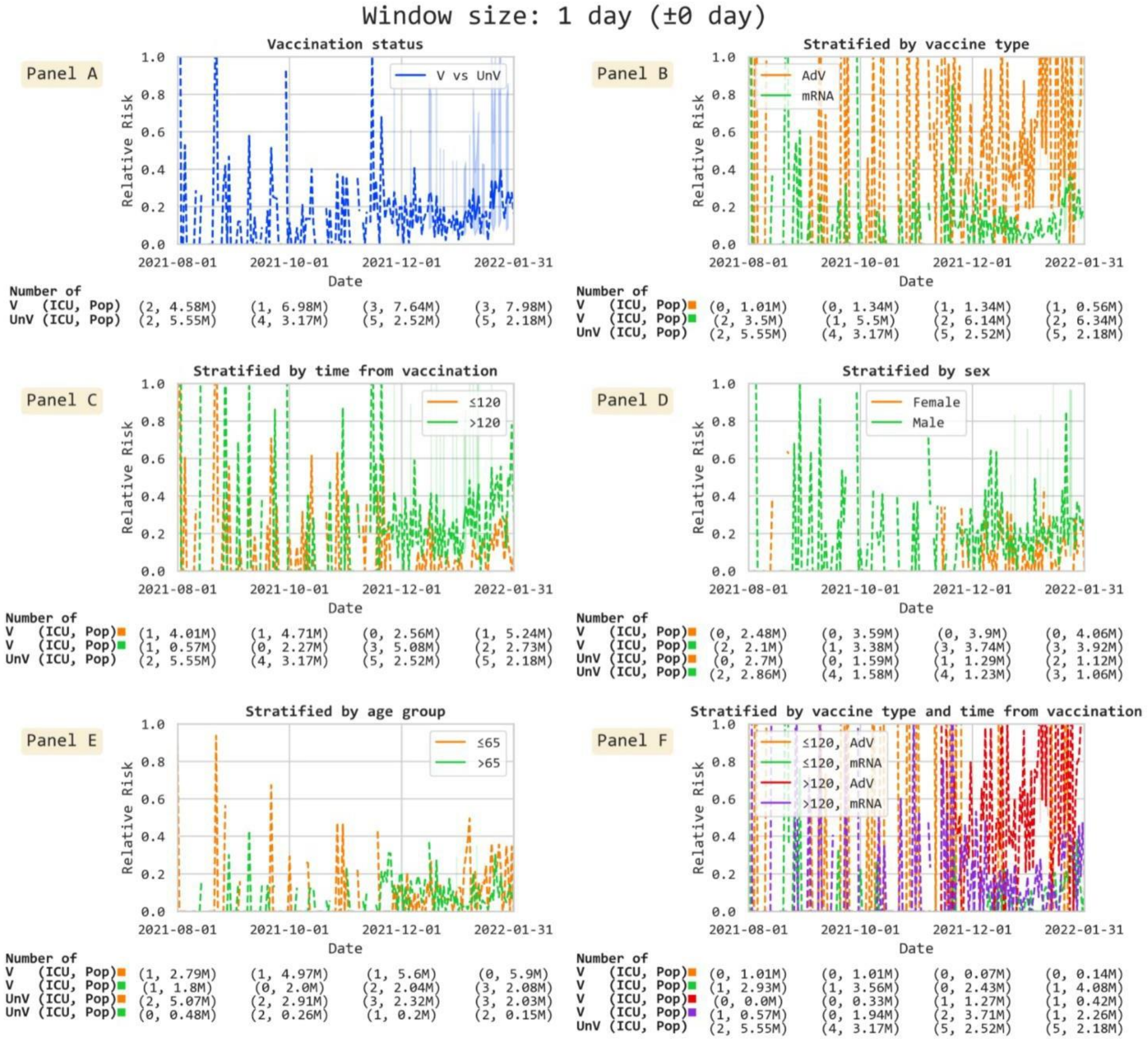

eFigure 2 shows the trend over time of the relative risk (RR) of being admitted to the ICU with COVID-19 pneumonia using a window size of 1 day (without any window function). Admissions are stratified by vaccination status (panel A), vaccine type (panel B), time from vaccination (panel C), sex (panel D), age (panel E), and a combination of vaccine type and time from vaccination (panel F).

The dashed lines represent less than 5 vaccinated or unvaccinated patients admitted in ICU.
Abbreviations. V, vaccinated; UnV, unvaccinated; AdV, vaccinated with adenoviral vaccines; mRNA, vaccinated with mRNA vaccines; ≤120, time from the last administered vaccine dose less than or equal to 120 days; >120, time from the last administered vaccine dose more than 120 days; ≤65, individuals and patients with age below or equal to 65-year-old; >65, individuals and patients with age above 65-year-old; ICU, number of patients admitted to the ICU; Pop, number of individuals in Lombardy.

© 2022 Grasselli G et al. *JAMA Network Open.*

eFigure 3. Relative risks with window size of 15 days.

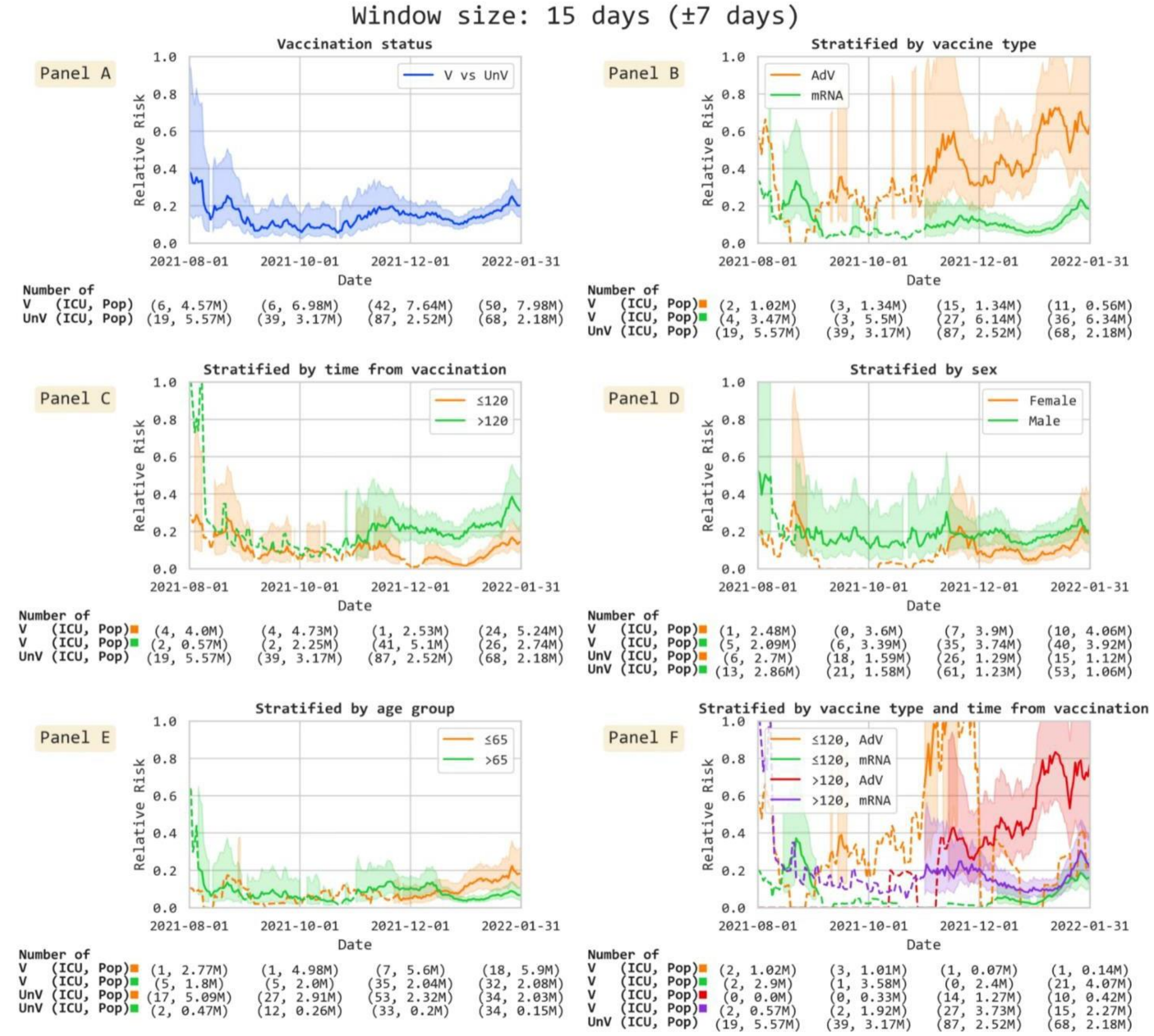

eFigure 3 shows the trend over time of the relative risk (RR) of being admitted to the ICU with COVID-19 pneumonia using a window size of 15 days. Admissions are stratified by vaccination status (panel A), vaccine type (panel B), time from vaccination (panel C), sex (panel D), age (panel E), and a combination of vaccine type and time from vaccination (panel F).

The dashed lines represent less than 5 vaccinated or unvaccinated patients admitted in ICU.
Abbreviations. V, vaccinated; UnV, unvaccinated; AdV, vaccinated with adenoviral vaccines; mRNA, vaccinated with mRNA vaccines; ≤120, time from the last administered vaccine dose less than or equal to 120 days; >120, time from the last administered vaccine dose more than 120 days; ≤65, individuals and patients with age below or equal to 65-year-old; >65, individuals and patients with age above 65-year-old; ICU, number of patients admitted to the ICU; Pop, number of individuals in Lombardy.

© 2022 Grasselli G et al. *JAMA Network Open.*

eFigure 4. Relative risks with window size of 29 days.

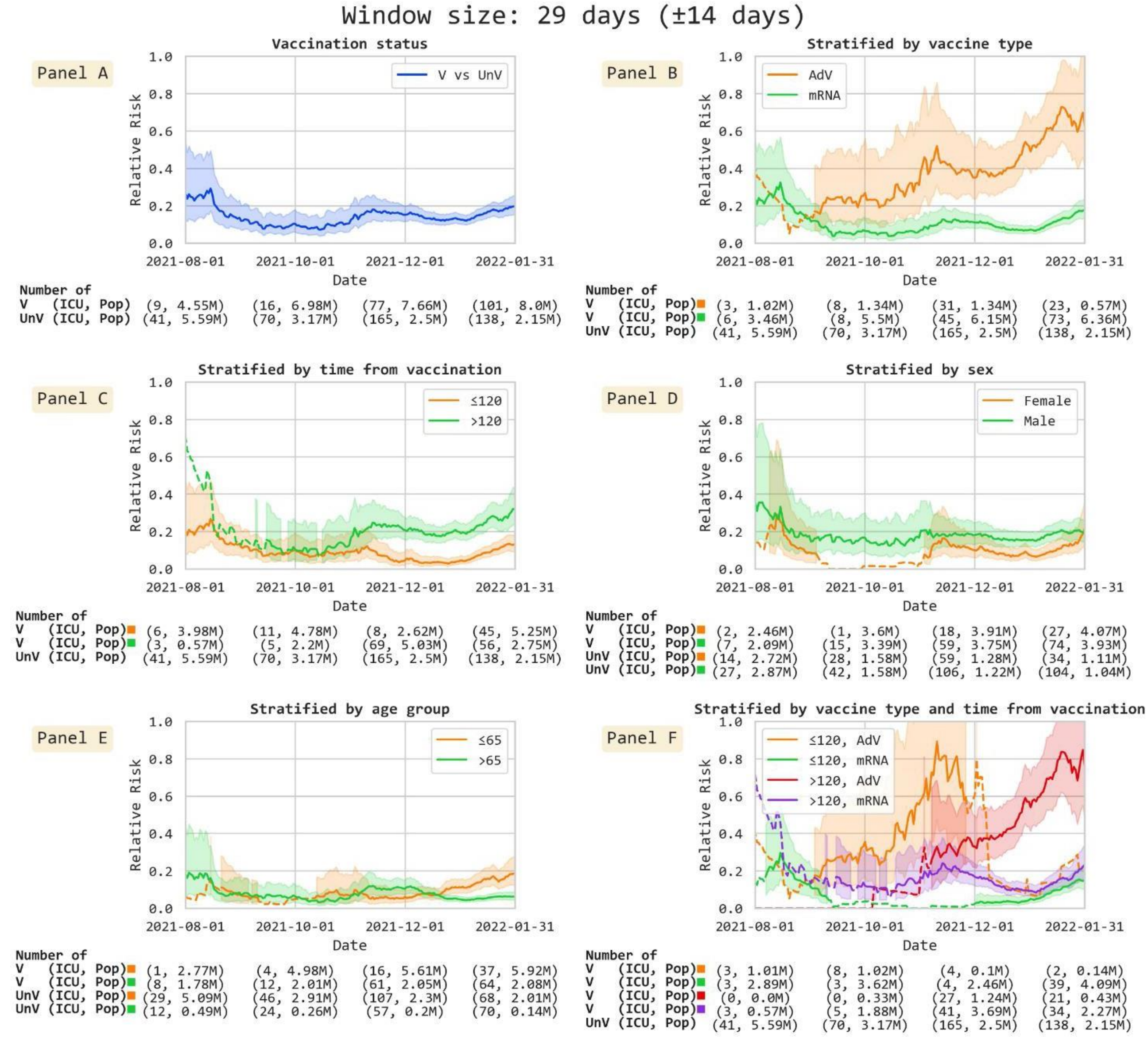

eFigure 4 shows the trend over time of the relative risk (RR) of being admitted to the ICU with COVID-19 pneumonia using a window size of 29 days (as in the Figure 2 of the main manuscript). Admissions are stratified by vaccination status (panel A), vaccine type (panel B), time from vaccination (panel C), sex (panel D), age (panel E), and a combination of vaccine type and time from vaccination (panel F).

The dashed lines represent less than 5 vaccinated or unvaccinated patients admitted in ICU.

Abbreviations. V, vaccinated; UnV, unvaccinated; AdV, vaccinated with adenoviral vaccines; mRNA, vaccinated with mRNA vaccines; ≤120, time from the last administered vaccine dose less than or equal to 120 days; >120, time from the last administered vaccine dose more than 120 days; ≤65, individuals and patients with age below or equal to 65-year-old; >65, individuals and patients with age above 65-year-old; ICU, number of patients admitted to the ICU; Pop, number of individuals in Lombardy.

© 2022 Grasselli G et al. *JAMA Network Open.*

eFigure 5. Relative risks with window size of 43 days.

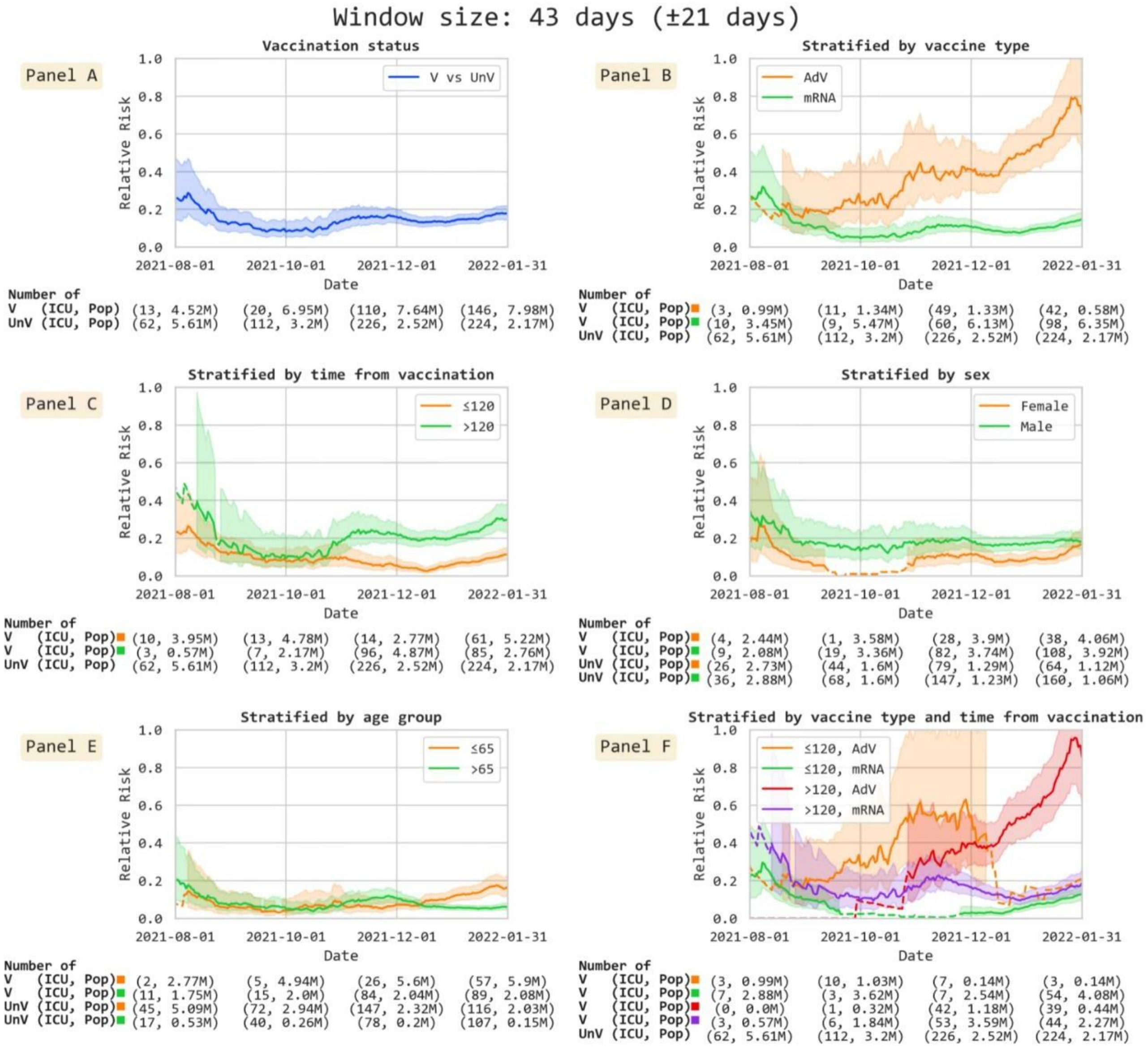

eFigure 5 shows the trend over time of the relative risk (RR) of being admitted to the ICU with COVID-19 pneumonia using a window size of 43 days. Admissions are stratified by vaccination status (panel A), vaccine type (panel B), time from vaccination (panel C), sex (panel D), age (panel E), and a combination of vaccine type and time from vaccination (panel F).
The dashed lines represent less than 5 vaccinated or unvaccinated patients admitted in ICU.
Abbreviations. V, vaccinated; UnV, unvaccinated; AdV, vaccinated with adenoviral vaccines; mRNA, vaccinated with mRNA vaccines; ≤120, time from the last administered vaccine dose less than or equal to 120 days; >120, time from the last administered vaccine dose more than 120 days; ≤65, individuals and patients with age below or equal to 65-year-old; >65, individuals and patients with age above 65-year-old; ICU, number of patients admitted to the ICU; Pop, number of individuals in Lombardy.

eFigure 6. Vaccination rate stratified by type of vaccine in Lombardy

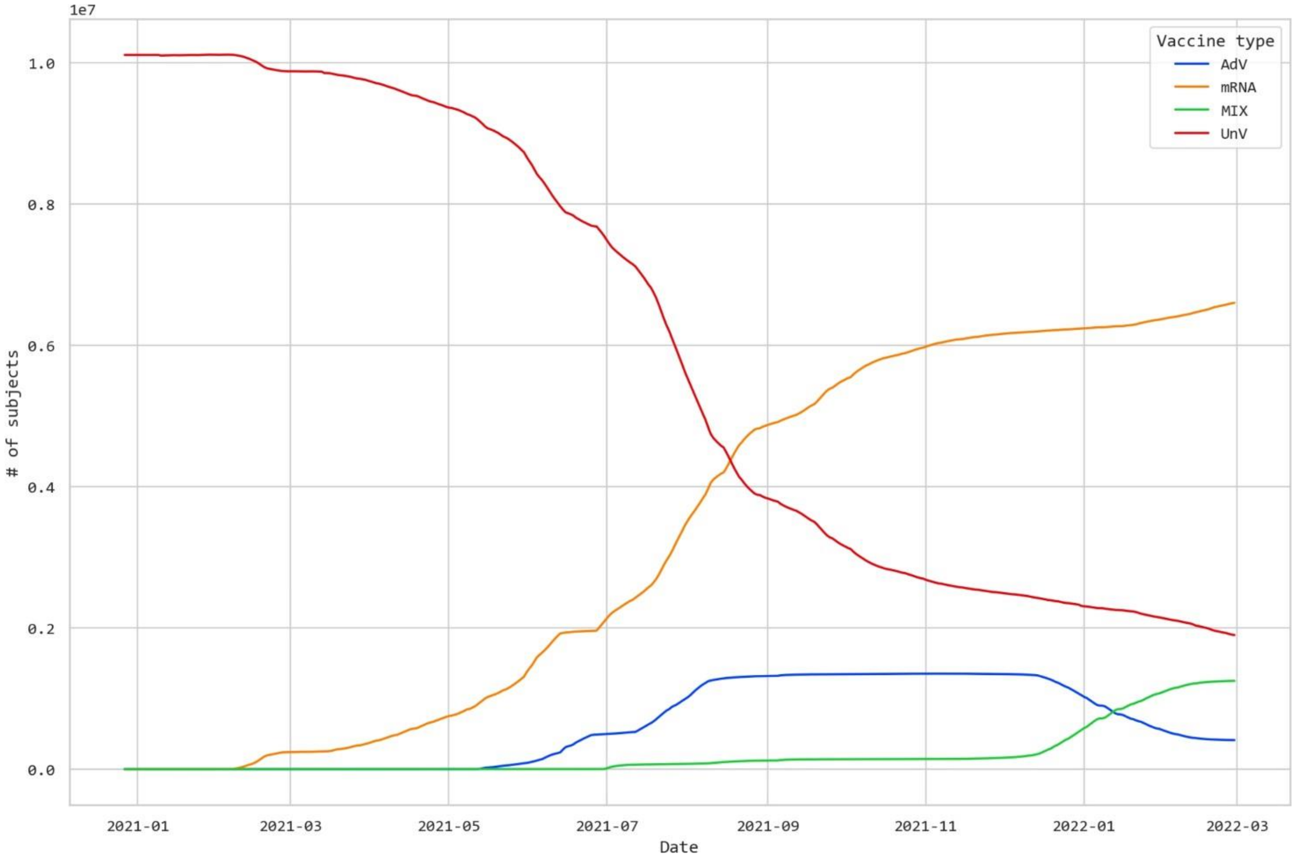

eFigure 6 shows the number of vaccinated and unvaccinated individuals in Lombardy over time. The red lines represent unvaccinated individuals, blue lines represent individuals vaccinated with AdV vaccines, orange lines represent individuals vaccinated with mRNA vaccines, green lines represent individuals vaccinated with mixed vaccines (AdV plus mRNA vaccines).

© 2022 Grasselli G et al. *JAMA Network Open.*

eFigure 7. Vaccination rate stratified by booster dose and type of vaccine in Lombardy

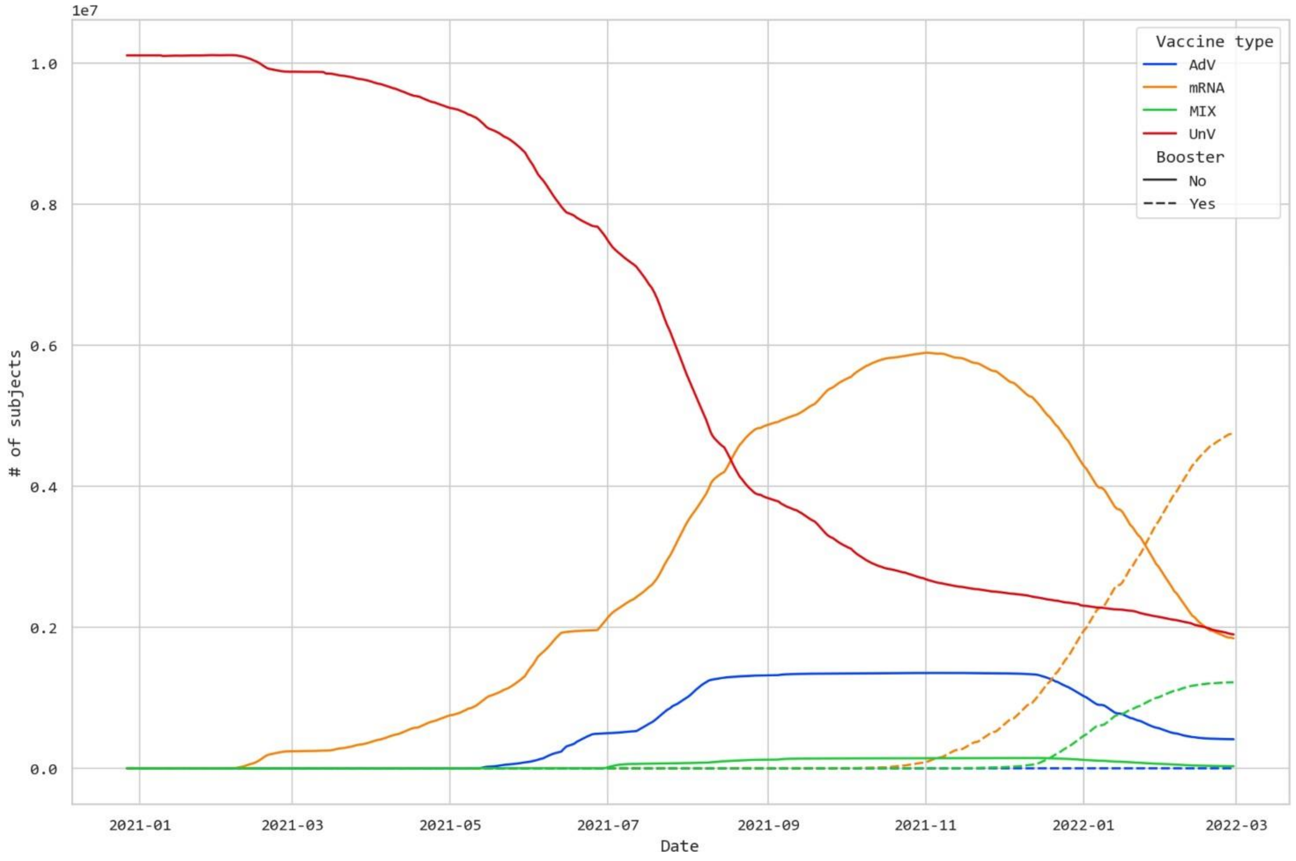

eFigure 7 shows the number of vaccinated and unvaccinated individuals in Lombardy over time. The red line represents unvaccinated individuals, blue lines represent individuals vaccinated with AdV vaccines, orange lines represent individuals vaccinated with mRNA vaccines, green lines represent individuals vaccinated with mixed vaccines (AdV and mRNA vaccines). Solid lines represent individuals that did not receive the booster dose, while dashed lines represent individual that received the booster dose.

© 2022 Grasselli G et al. *JAMA Network Open.*

eFigure 8. Vaccination rate by age and type of vaccine in Lombardy

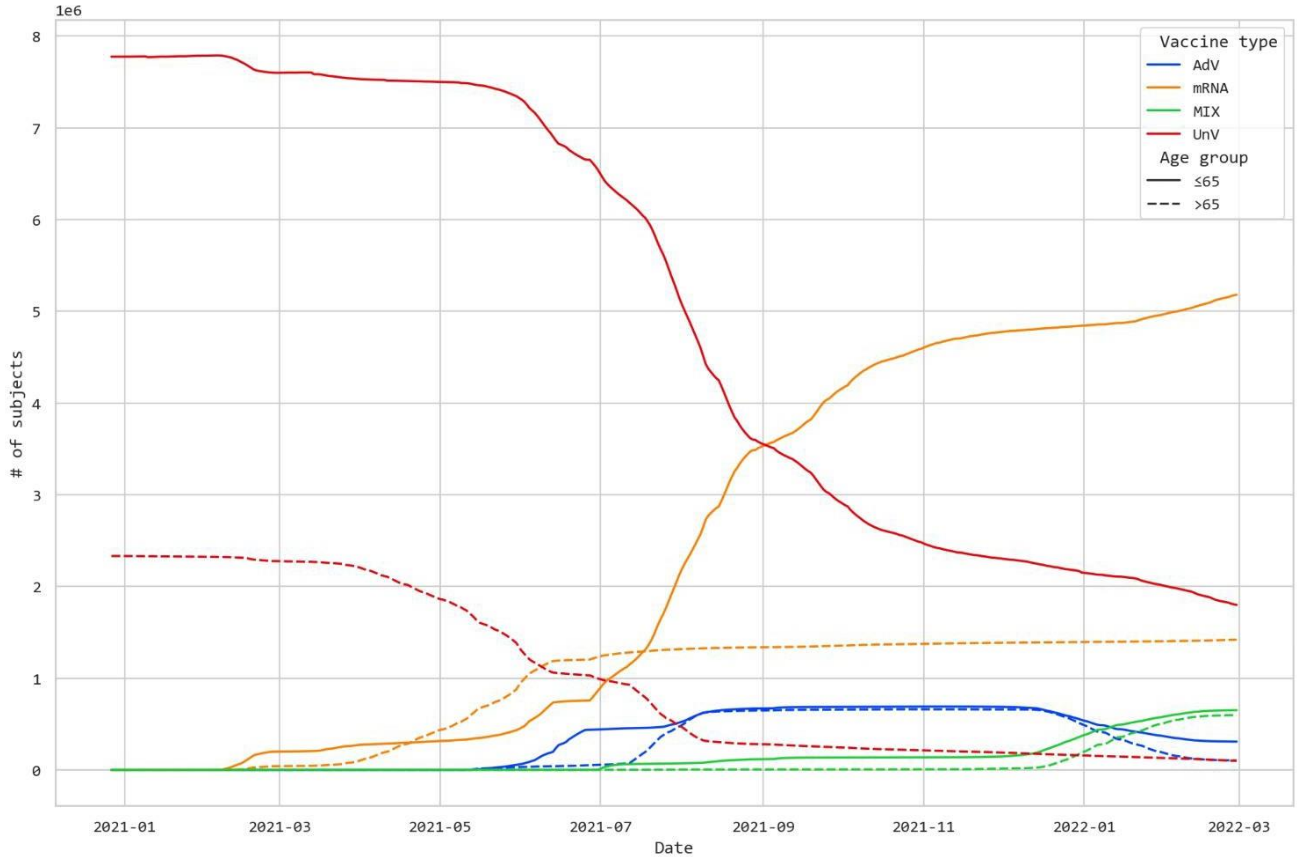

eFigure 8 shows the number of vaccinated and unvaccinated individuals in Lombardy over time. The red lines represent unvaccinated individuals, blue lines represent individuals vaccinated with AdV vaccines, orange lines represent individuals vaccinated with mRNA vaccines, green lines represent individuals vaccinated with mixed vaccines (AdV plus mRNA vaccines). Solid lines represent individuals with ages lower or equal to 65 years, while dashed lines represent individuals with age higher than 65 years.

© 2022 Grasselli G et al. *JAMA Network Open.*

eFigure 9. Vaccination rate by sex and type of vaccine in Lombardy.

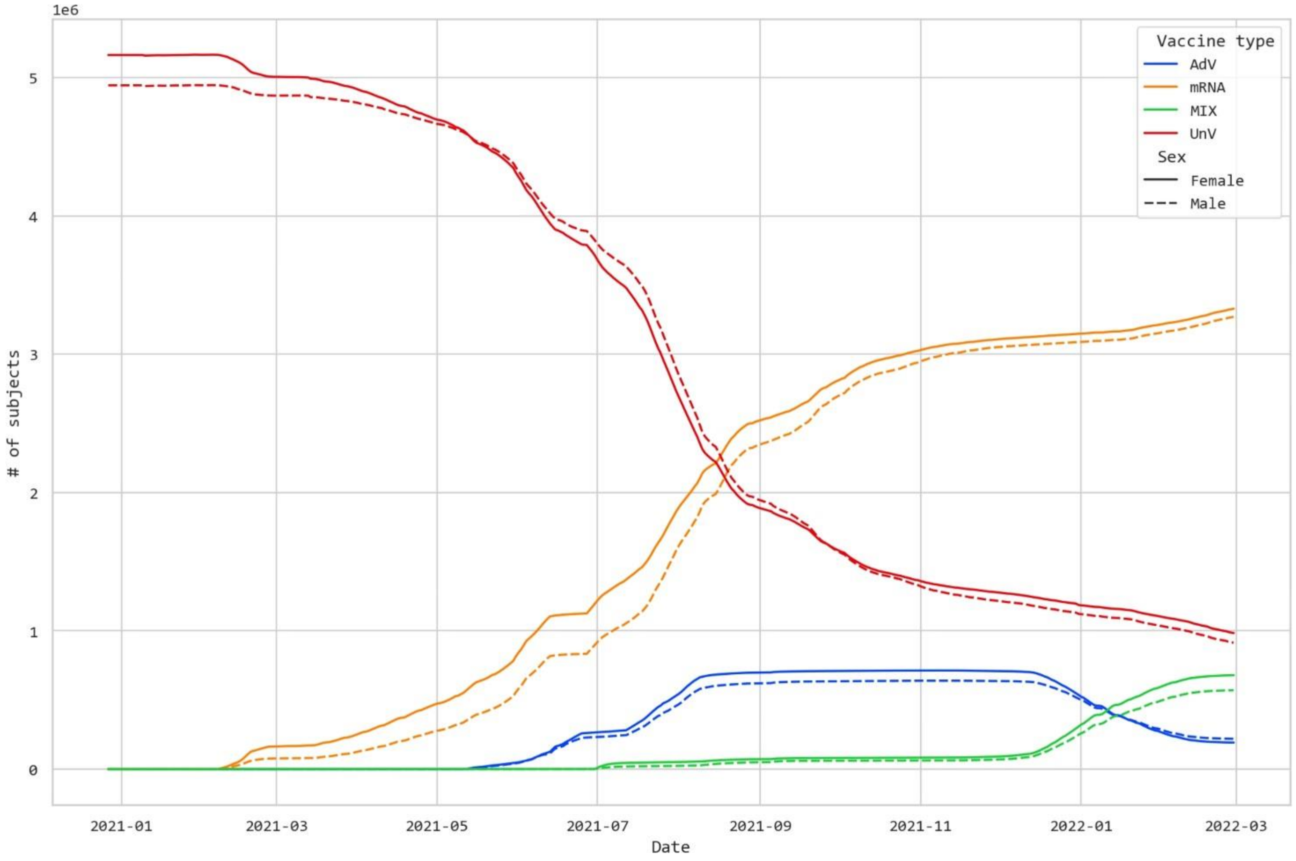

eFigure 9 shows the number of vaccinated and unvaccinated individuals in Lombardy over time. The red lines represent unvaccinated individuals, blue lines represent individuals vaccinated with AdV vaccines, orange lines represent individuals vaccinated with mRNA vaccines, green lines represent individuals vaccinated with mixed vaccines (AdV plus mRNA vaccines). Solid lines represent females, while dashed lines males.

© 2022 Grasselli G et al. *JAMA Network Open.*

eTable 4. Patients admitted to ICU, December 27, 2021 – January 31, 2022.

Characteristics	All	Unvaccinated	Vaccinated	P Unvaccinated vs. Vaccinated	mRNA	AdV	P Type of vaccine	P Unvaccinated vs mRNA	P Unvaccinated vs AdV	P AdV vs mRNA
N	5269	4923	346		198	148				
Age, years	66 [58-73]	66 [57-73]	70 [64-75]	<0.001	69 [62-74]	71 [66-75]	<0.001	0.001	<0.001	0.08
N	5238	4892	346		198	148				
Female	1581 (30.01%)	1505 (30.57%)	76 (21.97%)	0.001	55 (27.78%)	21 (14.19%)	<0.001	0.99	<0.001	0.008
N	5269	4923	346		198	148				
**Comorbidities**										
Number of comorbidities	1 [0-2]	1 [0-2]	2 [1-3]	<0.001	2 [1-4]	1 [1-2]	<0.001	<0.001	0.002	<0.001
N	5269	4923	346		198	148				
Hypertension	2319 (43.99%)	2108 (42.82%)	211 (60.98%)	<0.001	123 (62.12%)	88 (59.46%)	<0.001	<0.001	<0.001	0.99
N	5269	4923	346		198	148				
Hypercholesterolemia	910 (17.27%)	821 (16.68%)	89 (25.72%)	<0.001	53 (26.77%)	36 (24.32%)	<0.001	<0.001	0.04	0.99
N	5269	4923	346		198	148				
Heart disease	964 (18.30%)	876 (17.79%)	88 (25.43%)	<0.001	59 (29.80%)	29 (19.59%)	<0.001	<0.001	0.99	0.09
N	5269	4923	346		198	148				
Diabetes (type 2)	865 (16.42%)	775 (15.74%)	90 (26.01%)	<0.001	60 (30.30%)	30 (20.70%)	<0.001	<0.001	0.4125	0.11
N	5269	4923	346		198	148				
COPD	208 (3.95%)	193 (3.92%)	15 (4.34%)	0.7017	6 (3.03%)	9 (6.08%)	0.3286	NA	NA	NA
N	5269	4923	346		198	148				
CKD	188 (3.57%)	153 (3.11%)	35 (10.12%)	<0.001	27 (13.64%)	8 (5.41%)	<0.001	<0.001	0.35	0.04

© 2022 Grasselli G et al. *JAMA Network Open.*

N	5269	4923	346		198	148				
Liver disease	121 (2.30%)	102 (2.07%)	19 (5.49%)	<0.001	17 (8.59%)	2 (1.35%)	<0.001	<0.001	0.99	0.01
N	5269	4923	346		198	148				
Malignancy	567 (10.76%)	493 (10.01%)	74 (21.39%)	<0.001	56 (28.28%)	18 (12.16%)	<0.001	<0.001	0.99	0.0009
N	5269	4923	346		198	148				
No comorbidities	2023 (38.39%)	1959 (39.79%)	64 (18.50%)	<0.001	31 (15.66%)	33 (22.30%)	<0.001	<0.001	<0.001	0.35
N	5269	4923	346		198	148				
Other	994 (18.87%)	889 (18.06%)	105 (30.35%)	<0.001	83 (41.92%)	22 (14.86%)	<0.001	<0.001	0.96	<0.001
N°	5269	4923	346		198	148				
**Immunodepression**										
Solid organ transplantation	53 (1.01%)	35 (0.71%)	18 (5.20%)	<0.001	18 (9.09%)	0 (0.00%)	<0.001	<0.001	0.99	<0.001
N	5269	4923	346		198	148				
Autoimmune disease	217 (4.12%)	193 (3.92%)	24 (6.94%)	0.006	22 (11.11%)	2 (1.35%)	<0.001	<0.001	0.38	<0.001
N	5269	4923	346		198	148				
Chronic immunosuppressor treatment	1344 (25.51%)	1192 (24.21%)	152 (43.93%)	<0.001	109 (55.05%)	43 (29.05%)	<0.001	<0.001	0.53	<0.001
N	5269	4924	345		197	148				
**Disease chronology**										
Time from last dose to ICU, days	NA	NA	163 [120-195]	NA	163.5 [107-206]	162.5 [136-185]	0.48	NA	NA	NA
N	NA	NA	346		198	148				
Time from hospital admission to ICU admission, days	3 [1-6]	3 [1-6]	3 [0-6]	0.01	2 [0-6]	3 [1-6]	0.01	0.008	0.89	0.24
N	4719	4379	340		193	147				
Time from diagnosis to ICU admission, days	6 [3-10]	6 [3-10]	6 [2-9]	0.009	5 [1-10]	6 [3-9]	0.008	0.006	0.87	0.20

N	4732	4386	346		198	148				
**Respiratory support at ICU admission**										
Invasive ventilation	2911 (62.05%)	2696 (61.79%)	215 (65.55%)	0.18	113 (60.75%)	102 (71.83%)	0.05	0.99	0.05	0.11
N	4691	4363	328		186	142				
NIV	593 (12.62%)	553 (12.65%)	40 (12.20%)	0.81	26 (13.98%)	14 (9.86%)	0.52	NA	NA	NA
N	4700	4372	328		186	142				
CPAP	916 (19.49%)	859 (19.65%)	57 (17.38%)	0.32	34 (18.28%)	23 (16.20%)	0.54	NA	NA	NA
N	4700	4372	328		186	142				
ECMO	15 (0.43%)	14 (0.43%)	1 (0.39%)	0.92	0 (0.0%)	1 (0.85%)	0.48	NA	NA	NA
N	3521	3262	259		141	118				
Prone Position	923 (24.03%)	854 (23.98%)	69 (24.73%)	0.78	30 (20.00%)	39 (30.23%)	0.13	NA	NA	NA
N	3841	3562	279		150	129				
**Respiratory parameters**										
PaO_2, mmHg	83 [70-102]	82 [70-101]	87 [73-106]	0.03	87.5 [72.5-110]	87 [75-100]	0.10	NA	NA	NA
N	3575	3318	257		140	117				
FiO_2, %	70 [60-90]	75 [60-95]	65 [60-80]	<0.001	60 [60-80]	70 [60-90]	<0.001	<0.001	0.3935	0.1512
N	3709	3439	270		148	122				
PaO_2/FiO_2, mmHg	120 [89-164]	120 [89-163]	133 [98-176]	0.002	138 [95-188]	133 [99-163]	0.01	0.01	0.27	0.52
N	3569	3313	256		139	117				
PEEP, cmH_2O	10 [8-12]	10 [8-12]	10 [8-12]	0.97	10 [8-12]	10 [9-12]	0.97	NA	NA	NA
N	3611	3346	265		146	119				
**Outcomes**										

© 2022 Grasselli G et al. *JAMA Network Open.*

ICU-mortality, %	1868 (35.45%)	1733 (35.20%)	135 (39.02%)	0.15	77 (38.89%)	58 (39.19%)	0.36	NA	NA	NA
N	5269	4923	346		198	148				
ICU length of stay, days	14 [7-25]	14 [7-25]	13 [7-24]	0.43	11 [6-23]	16 [8.5-24.5]	0.05	0.09	0.45	0.05
N	5269	4923	346		198	148				
Hospital length of stay, days	28 [18-41]	28 [18-42]	24 [16-35]	<0.001	23[15-35]	26 [18-36]	<0.001	<0.001	0.09	0.29
N	4719	4379	340		193	147				
Mechanical Ventilation during ICU stay, days	10 [3-22]	10 [3-22]	10 [3-22]	0.53	8 [1-21]	14 [6-24]	0.02	0.41	0.04	0.02
N	4959	4617	342		194	148				
Hospital mortality	1983 (40.34%)	1828 (39.97%)	155 (45.19%)	0.06	92 (46.94%)	63 (42.86%)	0.12	NA	NA	NA
N	4916	4573	343		196	147				
Invasive ventilation ICU	3987 (80.40%)	3698 (80.10%)	289 (84.50%)	0.05	155 (79.90%)	134 (90.54%)	0.007	0.99	0.005	0.02
N	4959	4617	342		194	148				
ECMO	166 (4.11%)	161 (4.30%)	5 (1.72%)	0.0329	2 (1.26%)	3 (2.27%)	0.0962	NA	NA	NA
N	4039	3748	291		159	132				
Prone Position	3162 (72.39%)	2965 (72.99%)	197 (64.38%)	0.001	96 (56.47%)	101 (74.26%)	<0.001	<0.001	0.99	0.004
N	4368	4062	306		170	136				

eTable 4. Baseline characteristics and outcomes in vaccinated and unvaccinated patients admitted to ICU from December 27, 2021 to January 31, 2022. Categorical variables are reported as frequencies (percentages) and continuous variables as the median and interquartile range (IQR). Abbreviations: AdV, adenovirus vaccines; COPD, chronic obstructive pulmonary disease; CKD, chronic kidney disease; ICU, intensive care unit; NIV, non-invasive ventilation; CPAP, continuous positive airway pressure; ECMO, extracorporeal membrane oxygenation; PaO_2, arterial partial pressure of oxygen; FiO_2, fraction of inspiratory oxygen; PEEP, positive end-expiratory pressure. NA not applicable.

"P Type of vaccine" column refers to the comparison among the three groups of patients: unvaccinated, vaccinated with mRNA and vaccinated with AdV.

© 2022 Grasselli G et al. *JAMA Network Open.*

eTable 5. Genotypes frequency in ICU.

Date range	All ICU patients N	Tot patients with genotyping N (%)	Alpha N (%)	Delta N (%)	Omicron N (%)	Other N (%)
27/12/2020-31/1/2022	5269	957[@] (18.2%)	351 (36.7)	483 (50.5%)	58 (6.1%)	84 (8.8%)
27/12/2020-31/7/2021	4106	417[§] (10.2%)	350 (83.9%)	29 (7.0%)	0 (0.0%)	44 (10.6%)
1/8/2021 – 15/12/2021	553	309* (55.9%)	0 (0%)	297 (96.1%)	0 (0%)	22 (7.1%)
16/12/2021 – 31/1/2022	610	231[&] (38%)	1 (0.4%)	157 (68.0%)	58 (25.1%)	18 (7.8%)
1/8/2021 – 31/1/2022	1163	540[#] (46.4%)	1 (0.2%)	454 (84.1%)	58 (10.7%)	0 (0%)

eTable 5 indicates the number of available genotypes frequency in the different date ranges considered in the paper. Interestingly, some patients demonstrated more than 1 genotype: *:10 both delta and other; #:2 both delta and omicron, 10 both delta and other, 1 both alpha and delta; @: 2 both delta and omicron, 15 both delta and other, 1 both alpha and delta, 1 both alpha and other; §: 5 both delta and other, 1 both alpha and other; &: 2 both delta and omicron, 1 both alpha and delta.

eFigure 10. Number of ICU admissions according to vaccination status.

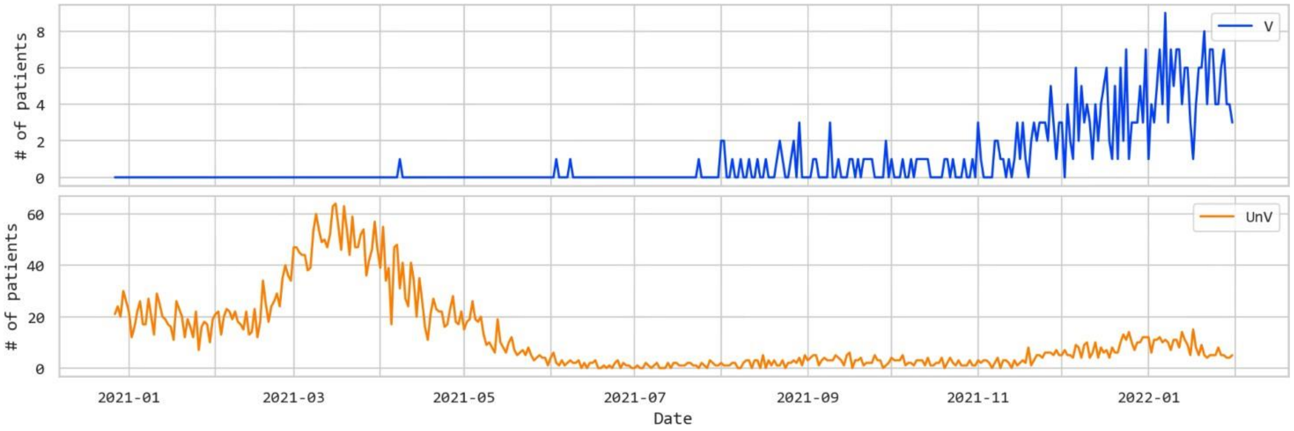

eFigure 10 shows the distribution of patients admitted to the ICU over time. The upper panel (blue line) represents vaccinated patients (V); the lower panel (orange line) represents unvaccinated patients (UnV).

© 2022 Grasselli G et al. *JAMA Network Open.*

eFigure 11. Prevalence of SARS-CoV-2 variants.

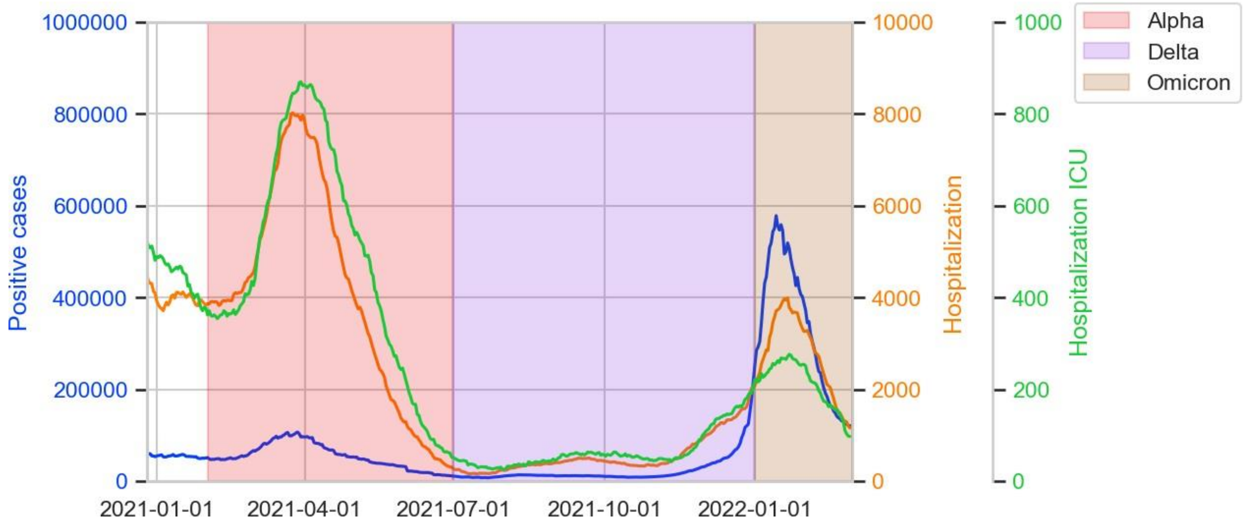

eFigure 11 shows the number of the current positive cases (hospitalized patients and home confinement) in blue line, the number of hospitalized patients in orange line; and number of the hospitalized patients in ICU in green line. The filled colored in the background show the dominant Variant of Concern (VOC) of the given period. The pink area is for the Alpha variant, light purple is for Delta variant and the almond area is for Omicron variant.

eFigure 12. Number of ICU admissions according to SARS-CoV-2 variants.

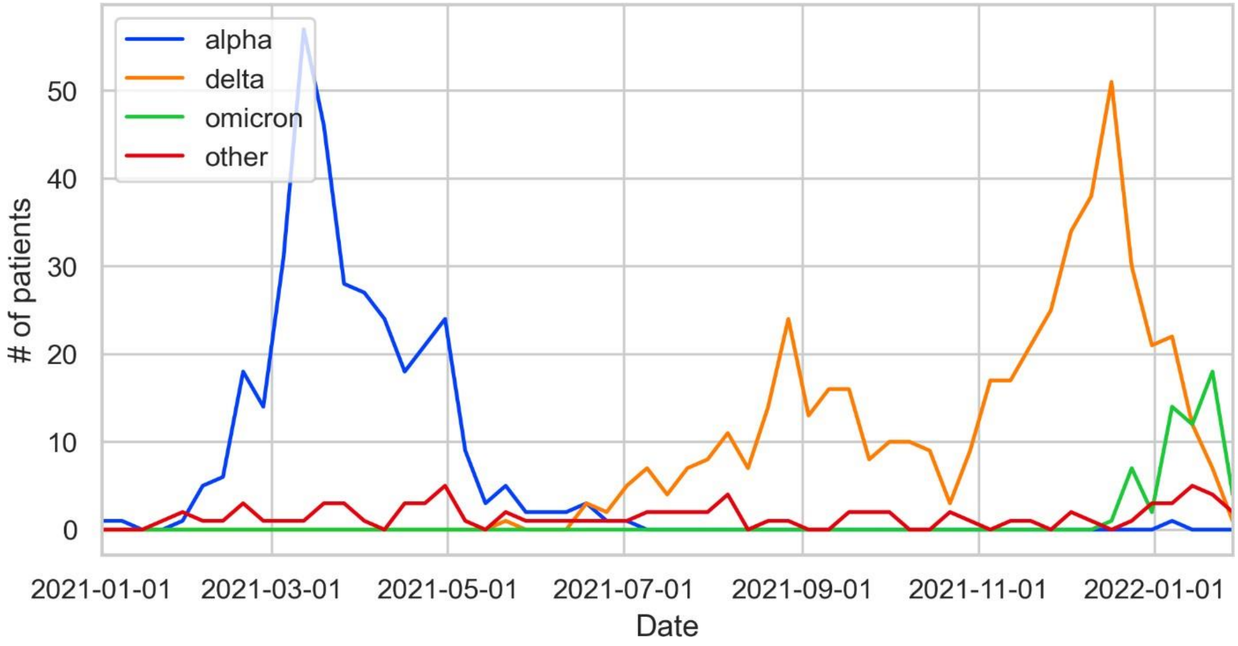

eFigure 12 shows the number of ICU admission stratified by genotypes of the variant (variant of concerns-VOCs). Blue line represents alpha variant, orange line represents delta variant, green line represents omicron variant and red line represent other variants.

© 2022 Grasselli G et al. *JAMA Network Open.*

eFigure 13. Prevalence of SARS-CoV-2 variants in ICU.

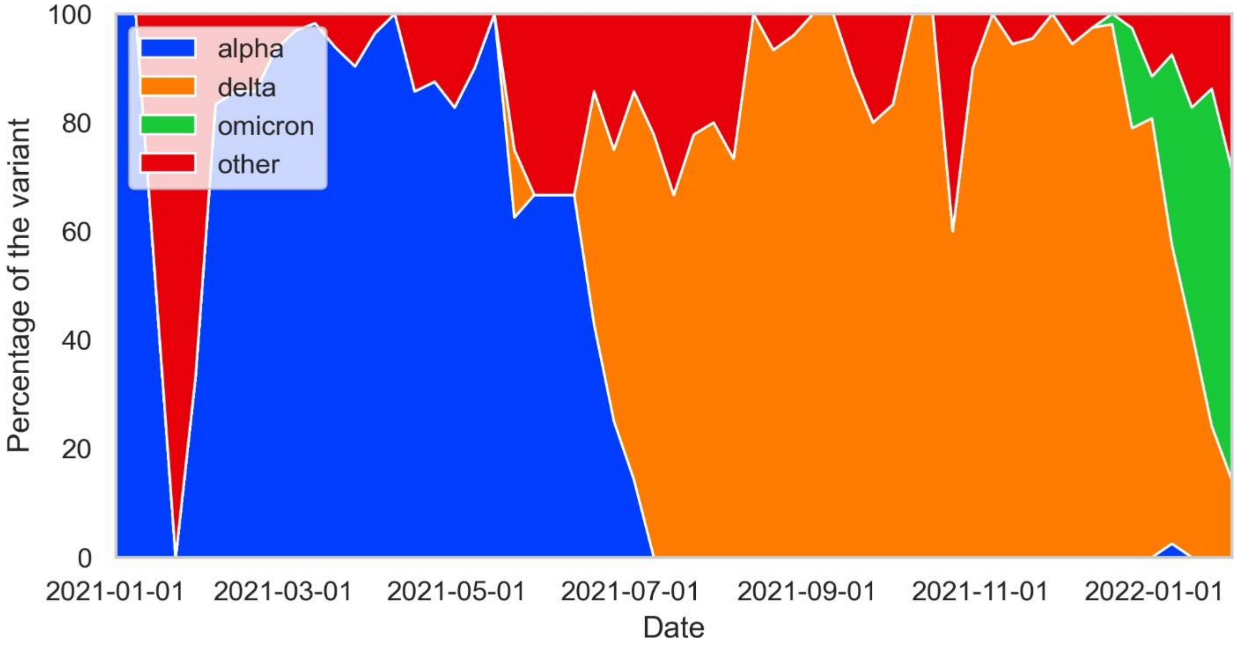

eFigure 13 shows the percentage of ICU admission stratified by genotypes of the variant (variant of concerns-VOCs). Blue color represents alpha variant, orange color represents delta variant, green color represents omicron variant and red color represents other variants.

© 2022 Grasselli G et al. *JAMA Network Open.*

eTable 6. Role of vaccination in preventing ICU admission

**Negative Binomial Models**	**IRR (95%CI)**
Female vs. male individuals (ref. Males) unadjusted	0.46 (0.40-0.53)
Female vs. male individuals (ref. Males) adjusted for vaccination	0.39 (0.35-0.45)
Female vs. male individuals (ref. Males) adjusted for vaccine type	0.33 (0.29-0.37)
≤ 65 vs. > 65-year-old (ref > 65y) unadjusted	0.31 (0.27-0.35)
≤ 65 vs. > 65-year-old (ref > 65y) adjusted for vaccination	0.14 (0.13-0.16)
≤ 65 vs. > 65-year-old (ref > 65y) adjusted for vaccine type	0.17 (0.15-0.19)
Vaccination (ref. No Vaccine) unadjusted	0.15 (0.13-0.17)
Vaccination (ref. No Vaccine) adjusted for sex	0.13 (0.11-0.15)
Vaccination (ref. No Vaccine) adjusted for age group	0.08 (0.07-0.09)
Vaccination (ref. No Vaccine) adjusted for age group and sex	0.07 (0.06-0.08)
Grouped by sex (ref. No Vaccine) unadjusted	
Males	0.18 (0.15-0.21)
Females	0.09 (0.08-0.11)
Grouped by age class (ref. No Vaccine) unadjusted	
≤65-year-old	0.10 (0.08-0.11)
>65-year-old	0.07 (0.06-0.09)
Type of vaccine (ref. No Vaccine) unadjusted	
AdV	0.43 (0.36-0.52)
mRNA	0.10 (0.08-0.12)
Type of vaccine (ref. No Vaccine) adjusted for age group and sex	
AdV	0.15 (0.13-0.16)
mRNA	0.06 (0.05-0.06)
Type of vaccine (ref. mRNA) unadjusted	
AdV	4.36 (3.61-5.25)
No Vaccine	10.02 (8.34-12.04)
Type of vaccine (ref. mRNA) adjusted for age group and sex	
AdV	2.66 (2.38-2.98)
No Vaccine	18.13 (16.31-20.14)
Time from vaccination (ref. No Vaccine) unadjusted	
≤ 120 days	0.07 (0.06-0.09)
> 120 days	0.22 (0.18-0.25)
Type of vaccine and time from vaccination (ref. No Vaccine) unadjusted	
Adenovirus ≤120 days	0.22 (0.18-0.27)
mRNA ≤120 days	0.06 (0.05-0.07)
Adenovirus > 120 days	0.54 (0.44-0.65)
mRNA > 120 days	0.15 (0.12-0.18)
Vaccination type and time from vaccination (ref. No Vaccine) adjusted for sex and age	
Adenovirus ≤120 days	0.06 (0.05-0.07)
mRNA ≤120 days	0.03 (0.03-0.04)
Adenovirus > 120 days	0.21 (0.19-0.24)
mRNA > 120 days	0.09 (0.08-0.10)

eTable 6 reports the results of multivariable generalized linear models with a negative binomial distribution and a log offset of population to account for different observations over time. Association was estimated using the Incidence Rate Ratio (IRR) and its related 95% confidence interval (CI). All p-values were < .001.

© 2022 Grasselli G et al. *JAMA Network Open.*

eTable 7. Multivariable analysis - Comparison between OR and RR estimated by multivariable logistic and log-binomial model, respectively

		Multivariable logistic model		Multivariable log-binomial model	
	**Category**	**OR (95% CI)**	**p-value**	**RR (95% CI)**	**p-value**
**Outcome: ICU mortality***					
Age (years)	5-y increments	1.39 (1.24-1.56)	<.001	1.23 (1.15-1.33)	<.001
Sex	Female vs Male	1.64 (1.02-2.63)	0.04	1.42 (1.07-1.89)	0.02
Heart disease	Yes vs No	2.62 (1.40-4.90)	0.003	1.62 (1.21-2.18)	0.001
PaO_2/FiO_2 (mmHg)	20 mmHg increments	0.81 (0.73-0.89)	<.001	0.88 (0.84-0.93)	<.001
**Outcome: Hospital mortality°**					
Age (years)	5-y increments	1.46 (1.30-1.64)	<.001	1.24(1.18-1.32)	<.001
Heart disease	Yes vs No	1.85 (1.00-3.42)	0.05	1.40 (1.08-1.82)	0.02
PaO_2/FiO_2 (mmHg)	20 mmHg increments	0.88 (0.81-0.96)	<.001	0.93 (0.89-0.98)	0.005

* Multivariable models on ICU mortality were carried out on 454 individuals. Hosmer-Lemeshow test reported a p-value equal to 0.26.

° Multivariable models on hospital mortality were carried out on 454 individuals. Hosmer-Lemeshow test reported a p-value equal to 0.44.

Abbreviations. CI: confidence interval; OR: odds ratio; RR: relative risk.

Note. Predictors were defined using backward stepwise approach. The list od possible predictors was: age, sex, vaccination status, type of comorbidity (hypertension, heart disease, diabetes mellitus, chronic renal disease, liver disease, chronic obstructive pulmonary disease, malignancy), immunosuppressive therapy, number of comorbidities (categorized as 0, 1, 2 and >2), PaO_2/FiO_2 at ICU admission.

eTable 7 reports OR and RR estimates using multivariable logistic and log-binomial model, respectively.

© 2022 Grasselli G et al. *JAMA Network Open.*

eFigure 14. Relative risk according to the different types of vaccines.

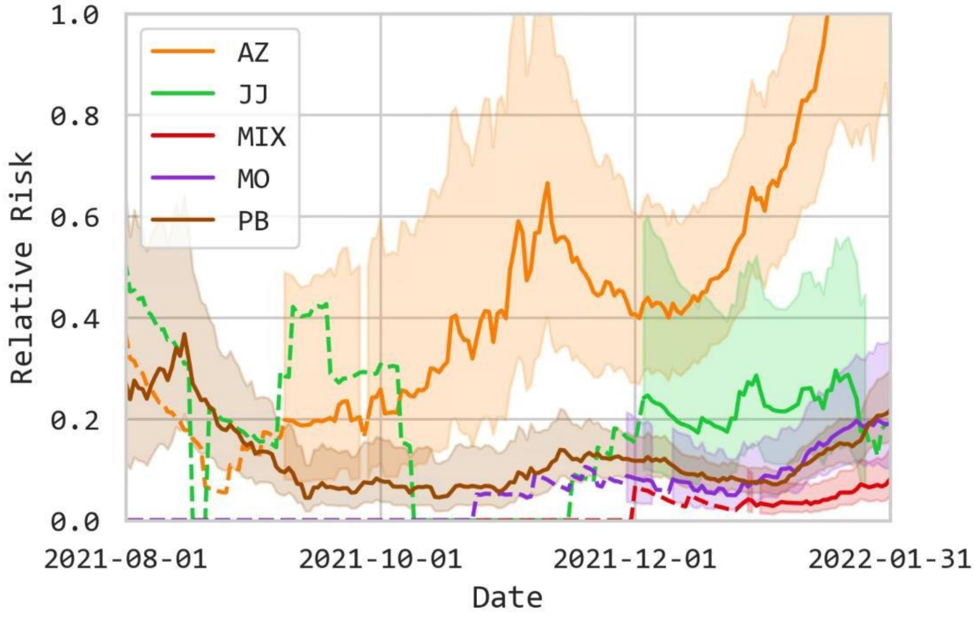

Number of				
V (ICU, Pop)	(2, 0.74M)	(6, 1.05M)	(28, 1.05M)	(19, 0.24M)
V (ICU, Pop)	(1, 0.27M)	(2, 0.29M)	(3, 0.3M)	(4, 0.33M)
V (ICU, Pop)	(0, 0.08M)	(0, 0.14M)	(1, 0.22M)	(14, 2.69M)
V (ICU, Pop)	(0, 0.43M)	(0, 0.85M)	(5, 0.92M)	(11, 0.9M)
V (ICU, Pop)	(6, 3.02M)	(8, 4.65M)	(40, 5.17M)	(53, 3.83M)
UnV (ICU, Pop)	(41, 5.59M)	(70, 3.17M)	(165, 2.5M)	(138, 2.15M)

eFigure 14 shows trend over time of the relative risk (RR) of being admitted to the ICU with COVID-19 pneumonia using a window size of 29 days. Admissions are stratified by vaccine type. The dashed lines represent less than 5 vaccinated or unvaccinated patients admitted in ICU. Abbreviations. V, vaccinated; UnV, unvaccinated; AdV, vaccinated with adenoviral vaccines; mRNA, vaccinated with mRNA vaccines; ≤120, time from the last administered vaccine dose less than or equal to 120 days; >120, time from the last administered vaccine dose more than 120 days; ≤65, individuals and patients with age below or equal to 65-year-old; >65, individuals and patients with age above 65-year-old; ICU, number of patients admitted to the ICU; Pop, number of individuals in Lombardy.

© 2022 Grasselli G et al. *JAMA Network Open.*

eTable 8. Number of missing and available data related to characteristics in Table 2.

Characteristics	All (N = 553)	Unvaccinated (N = 414)	Vaccinated (N = 139)	mRNA (N = 81)	AdV (N = 58)
Age					
Available	552	413	139	81	58
Missing	1	1	0	0	0
Sex					
Available	553	414	139	81	58
Missing	0	0	0	0	0
Comorbidities and Immunodepression data					
Available	553	414	139	81	58
Missing	0	0	0	0	0
**Disease chronology**					
Time from last dose to ICU	NA	NA			
Available			139	81	58
Missing			0	0	0
Time from hospital admission to ICU admission					
Available	498	363	135	78	57
Missing	55	51	4	3	1
Time from diagnosis to ICU admission					
Available	506	367	139	81	58
Missing	47	47	0	0	0
**Respiratory support at ICU admission**					
Invasive ventilation					
Available	532	398	134	77	57
Missing	21	16	5	4	1
NIV					
Available	534	400	134	77	57
Missing	19	14	5	4	1
CPAP					
Available	534	400	134	77	57
Missing	19	14	5	4	1
ECMO					
Available	464	356	108	61	47
Missing	89	49	31	20	11
Prone Position					
Available	476	362	114	63	51
Missing	77	52	25	18	7
**Respiratory parameters**					
PaO_2					
Available	455	351	104	59	45
Missing	98	63	35	22	13
FiO_2					
Available	470	358	112	63	49
Missing	83	56	24	18	6
PaO_2/FiO_2					
Available	454	351	103	58	45
Missing	99	63	36	23	13
PEEP					
Available	456	347	109	63	46
Missing	97	67	30	18	12
**Outcomes**					
ICU mortality					
Available	553	414	139	81	58
Missing	0	0	0	0	0
ICU length of stay					

© 2022 Grasselli G et al. *JAMA Network Open.*

Characteristics	All<br>(N = 553)	Unvaccinated<br>(N = 414)	Vaccinated<br>(N = 139)	mRNA<br>(N = 81)	AdV<br>(N = 58)
Available	553	414	139	81	58
Missing	0	0	0	0	0
Hospital mortality					
Available	512	375	137	80	57
Missing	502	39	2	1	1
Length of hospital stay					
Available	498	363	135	78	57
Missing	55	51	4	3	1
Mechanical Ventilation during ICU stay					
Available	545	408	137	79	58
Missing	8	6	2	2	0
Invasive ventilation ICU					
Available	545	408	137	79	58
Missing	8	6	2	2	0
ECMO					
Available	522	396	126	73	53
Missing	31	18	13	8	5
Prone Position					
Available	530	400	130	75	55
Missing	23	14	9	6	3

Abbreviations. NA not applicable

© 2022 Grasselli G et al. *JAMA Network Open.*
