# Supplementary material for: Association of COVID-19 Vaccinations With Intensive Care Unit Admissions and Outcome of Critically Ill Patients With COVID-19 Pneumonia in Lombardy, Italy
Source: JAMA Netw Open. 2022 Oct 27;5(10):e2238871. doi: 10.1001/jamanetworkopen.2022.38871 (PMC9614574; doi:10.1001/jamanetworkopen.2022.38871)
Supplement: Supplement 2. — Nonauthor Collaborators. COVID-19 Lombardy ICU Network [file jamanetwopen-e2238871-s002.pdf]

Supplemental Online Content: Nonauthor Collaborators
*First name, last name, and suffix (if applicable) are required and will appear in PubMed.

*Group Name(s): COVID-19 Lombardy ICU Network								
*First Name and Middle Initial(s)	*Last Name	*Suffix (eg, Jr, III)	Academic Degrees	Institution	Location (city, state/province, country)	Role or Contribution, eg, chair, principal investigator	Group (if more than 1 Group listed in the byline) and/or Subgroup (eg, Steering Committee)	
Roberto	Keim		MD	ASST Bergamo est Ospedale "Bologn	Seriate, Italy	Patient enrollment and	COVID-19 Lombardy ICU Network	
Federica	Vagginelli		MD	ASST Bergamo ovest Ospedale di Tre	Treviglio, Italy	Patient enrollment and	COVID-19 Lombardy ICU Network	
Antonello	Sala		MD	ASST Brianza Ospedale di Vimercate	Vimercate, Italy	Patient enrollment and	COVID-19 Lombardy ICU Network	
Guido	Merli		MD	ASST Crema Ospedale Maggiore di C	Crema, Italy	Patient enrollment and	COVID-19 Lombardy ICU Network	
Patrizia	Ruggeri		MD	ASST Cremona Ospedale di Cremona	Cremona, Italy	Patient enrollment and	COVID-19 Lombardy ICU Network	
Giorgio	Villani		MD	ASST Cremona Ospedale di Cremona	Cremona, Italy	Patient enrollment and	COVID-19 Lombardy ICU Network	
Mario	Riccio		MD	ASST Cremona Ospedale di Oglio Po	Casalmaggiore, Italy	Patient enrollment and	COVID-19 Lombardy ICU Network	
Elena	Zoia		MD	ASST Fatebenefratelli Sacco Children	Milan, Italy	Patient enrollment and	COVID-19 Lombardy ICU Network	
Antonio	Castelli		MD	ASST Fatebenefratelli Sacco Luigi Sac	Milan, Italy	Patient enrollment and	COVID-19 Lombardy ICU Network	
Riccardo	Colombo		MD	ASST Fatebenefratelli Sacco Luigi Sac	Milan, Italy	Patient enrollment and	COVID-19 Lombardy ICU Network	
Roberto	Rech		MD	ASST Fatebenefratelli Sacco Luigi Sac	Milan, Italy	Patient enrollment and	COVID-19 Lombardy ICU Network	
Paolo	Gnesin		MD	ASST Franciacorta Presidio Ospedalie	Chiari, italy	Patient enrollment and	COVID-19 Lombardy ICU Network	
Davide	Coppini		MD	ASST Garda Ospedale Civile "La Mem	Gavardo, Italy	Patient enrollment and	COVID-19 Lombardy ICU Network	
Nicola	Petrucci		MD	ASST Garda Ospedale di Desenzano (	Desenzano del Garda, Italy	Patient enrollment and	COVID-19 Lombardy ICU Network	
Benvenuto	Antonini		MD	ASST Garda Presidio Ospedaliero di M	Manerbio, Italy	Patient enrollment and	COVID-19 Lombardy ICU Network	
Luca	Landolfi		MD	ASST Garda Presidio Ospedaliero di M	Manerbio, Italy	Patient enrollment and	COVID-19 Lombardy ICU Network	
Fernando	Arnaiz		MD	ASST Grande Ospedale Metropolitan	Milan, Italy	Patient enrollment and	COVID-19 Lombardy ICU Network	
Clarissa	Forlini		MD	ASST Grande Ospedale Metropolitan	Milan, Italy	Patient enrollment and	COVID-19 Lombardy ICU Network	
Flavia	Stefanini		MD	ASST Grande Ospedale Metropolitan	Milan, Italy	Patient enrollment and	COVID-19 Lombardy ICU Network	
Antonio	Micucci		MD	ASST Lariana Ospedale di Sant'Anton	Cantù, Italy	Patient enrollment and	COVID-19 Lombardy ICU Network	
Jlenia Alessia	Marelli		MD	ASST Lariana Ospedale Sant'Anna	Como, Italy	Patient enrollment and	COVID-19 Lombardy ICU Network	
Francesco	Torresani		MD	ASST Lariana Ospedale Sant'Anna	Como, Italy	Patient enrollment and	COVID-19 Lombardy ICU Network	
Cristina	Panzeri		MD	ASST Lecco Ospedale di Lecco	Lecco, Italy	Patient enrollment and	COVID-19 Lombardy ICU Network	
Elena	Paolini		MD	ASST Lecco Ospedale di Lecco	Lecco, Italy	Patient enrollment and	COVID-19 Lombardy ICU Network	
Davide	Guzzon		MD	ASST Lecco Ospedale di Merate	Merate, Italy	Patient enrollment and	COVID-19 Lombardy ICU Network	
Laura	Maderna		MD	ASST Lodi Ospedale Maggiore di Lod	Lodi, Italy	Patient enrollment and	COVID-19 Lombardy ICU Network	
Gianluca	Russo		MD	ASST Lodi Ospedale Maggiore di Lod	Lodi, Italy	Patient enrollment and	COVID-19 Lombardy ICU Network	
Angela	Berselli		MD	ASST Mantova Ospedale Carlo Poma	Mantova, Italy	Patient enrollment and	COVID-19 Lombardy ICU Network	
Alberto	Biondo		MD	ASST Mantova Ospedale Carlo Poma	Mantova, Italy	Patient enrollment and	COVID-19 Lombardy ICU Network	
Claudio	Montalto		MD	ASST Mantova Ospedale Carlo Poma	Mantova, Italy	Patient enrollment and	COVID-19 Lombardy ICU Network	
Leonardo	Bianciardi		MD	ASST Mantova Ospedale Pieve di Cor	Borgo Mantovano, Italy	Patient enrollment and	COVID-19 Lombardy ICU Network	
Federica	Della Mura		MD	ASST Melegnano e Martesana Osped	Vizzolo Predabissi, Italy	Patient enrollment and	COVID-19 Lombardy ICU Network	

Supplemental Online Content: Nonauthor Collaborators
*First name, last name, and suffix (if applicable) are required and will appear in PubMed.

***First Name and Middle Initial(s)**	***Last Name**	***Suffix (eg, Jr, III)**	Academic Degrees	Institution	Location (city, state/province, country)	Role or Contribution, eg, chair, principal investigator	Group (if more than 1 Group listed in the byline) and/or Subgroup (eg, Steering Committee)	
Giovanni	Marino		MD	ASST Melegnano e Martesana Ospec	Vizzolo Predabissi, Italy	Patient enrollment and	COVID-19 Lombardy ICU Network	
Massimo	Zambon		MD	ASST Melegnano e Martesana Presid	Cernusco sul Naviglio, Italy	Patient enrollment and	COVID-19 Lombardy ICU Network	
Matteo	Subert		MD	ASST Melegnano e Martesana Presid	Melzo, Italy	Patient enrollment and	COVID-19 Lombardy ICU Network	
Giuseppe	Citerio		MD	ASST Monza Ospedale di Desio	Desio, Italy	Patient enrollment and	COVID-19 Lombardy ICU Network	
Maurizio	Saini		MD	ASST Monza Ospedale di Desio	Desio, Italy	Patient enrollment and	COVID-19 Lombardy ICU Network	
Roberto	Rona		MD	ASST Monza Ospedale San Gerardo	Monza, Italy	Patient enrollment and	COVID-19 Lombardy ICU Network	
Angelo	Pezzi		MD	ASST Nord Milano Ospedale "Edoard	Cinisello Balsamo, Italy	Patient enrollment and	COVID-19 Lombardy ICU Network	
Giovanni	Sabbatini		MD	ASST Nord Milano Ospedale "Edoard	Cinisello Balsamo, Italy	Patient enrollment and	COVID-19 Lombardy ICU Network	
Silvia	Paganini		MD	ASST Ovest Milanese Ospedale Nuov	Legnano (MI), Italy	Patient enrollment and	COVID-19 Lombardy ICU Network	
Virginia	Porta		MD	ASST Ovest Milanese Ospedale Nuov	Legnano (MI), Italy	Patient enrollment and	COVID-19 Lombardy ICU Network	
Alberto	Benigni		MD	ASST Papa Giovanni XXIII	Bergamo, Italy	Patient enrollment and	COVID-19 Lombardy ICU Network	
Ezio	Bonanomi		MD	ASST Papa Giovanni XXIII	Bergamo, Italy	Patient enrollment and	COVID-19 Lombardy ICU Network	
Fabrizio	Fabretti		MD	ASST Papa Giovanni XXIII	Bergamo, Italy	Patient enrollment and	COVID-19 Lombardy ICU Network	
Paolo	Gritti		MD	ASST Papa Giovanni XXIII	Bergamo, Italy	Patient enrollment and	COVID-19 Lombardy ICU Network	
Giorgio	Arachi		MD	ASST Pavia Ospedale Civile di Vogher	Voghera, Italy	Patient enrollment and	COVID-19 Lombardy ICU Network	
Maurizio	Raimondi		MD	ASST Pavia Ospedale Civile di Vogher	Voghera, Italy	Patient enrollment and	COVID-19 Lombardy ICU Network	
Alberto	Casazza		MD	ASST Pavia Ospedale di Vigevano	Vigevano, Italy	Patient enrollment and	COVID-19 Lombardy ICU Network	
Gianluca	De Filippi		MD	ASST Rhodense Presidio ospedaliero	Garbagnate Milanese, Italy	Patient enrollment and	COVID-19 Lombardy ICU Network	
Francesca	Piccoli		MD	ASST Rhodense Presidio ospedaliero	Rho, Italy	Patient enrollment and	COVID-19 Lombardy ICU Network	
Michele	Umbrello		MD	ASST Santi Paolo e Carlo Ospedale Sa	Milan, Italy	Patient enrollment and	COVID-19 Lombardy ICU Network	
Silvia	Coppola		MD	ASST Santi Paolo e Carlo Ospedale Sa	Milan, Italy	Patient enrollment and	COVID-19 Lombardy ICU Network	
Tommaso	Pozzi		MD	ASST Santi Paolo e Carlo Ospedale Sa	Milan, Italy	Patient enrollment and	COVID-19 Lombardy ICU Network	
Alberto	Corona		MD	ASST Valcamonica Ospedale di Esine	Esine, Italy	Patient enrollment and	COVID-19 Lombardy ICU Network	
Emanuele	Bossi		MD	ASST Valle Olona Ospedale di Gallara	Gallarate, Italy	Patient enrollment and	COVID-19 Lombardy ICU Network	
Stefano	Greco		MD	ASST Valle Olona Ospedale di Saronr	Saronno, Italy	Patient enrollment and	COVID-19 Lombardy ICU Network	
Alessandra	Besozzi		MD	ASST Valle Olona PO Busto Arsizio	Busto Arsizio, Italy	Patient enrollment and	COVID-19 Lombardy ICU Network	
Remo Daniel	Covello		MD	ASST Valle Olona PO Busto Arsizio	Busto Arsizio, Italy	Patient enrollment and	COVID-19 Lombardy ICU Network	
Mauro	Della Morte		MD	ASST Valtellina e Alto Lario Ospedale	Sondalo, Italy	Patient enrollment and	COVID-19 Lombardy ICU Network	
Fabio	Sangalli		MD	ASST Valtellina e Alto Lario Ospedale	Sondalo, Italy	Patient enrollment and	COVID-19 Lombardy ICU Network	
Dario	Gasberti		MD	Azienda Ospedaliera Ospedale di Cir	Varese, Italy	Patient enrollment and	COVID-19 Lombardy ICU Network	
Davide	Maraggia		MD	Azienda Ospedaliera Ospedale di Cir	Varese, Italy	Patient enrollment and	COVID-19 Lombardy ICU Network	
Alessandro	Motta		MD	Azienda Ospedaliera Ospedale di Cir	Varese, Italy	Patient enrollment and	COVID-19 Lombardy ICU Network	
Emiliano	Agosteo		MD	Clinica "San Carlo"	Paderno Dugnano, Italy	Patient enrollment and	COVID-19 Lombardy ICU Network	

*First name, last name, and suffix (if applicable) are required and will appear in PubMed.

***First Name and Middle Initial(s)**	***Last Name**	***Suffix (eg, Jr, III)**	Academic Degrees	Institution	Location (city, state/province, country)	Role or Contribution, eg, chair, principal investigator	Group (if more than 1 Group listed in the byline) and/or Subgroup (eg, Steering Committee)	
Lucia	Crottogini			Directorate General for Health, Loml	Milan, Italy	Patient enrollment anc	COVID-19 Lombardy ICU Network	
Olivia	Leoni			Directorate General for Health, Loml	Milan, Italy	Patient enrollment anc	COVID-19 Lombardy ICU Network	
Marcello	Tirani			Directorate General for Health, Loml	Milan, Italy	Patient enrollment anc	COVID-19 Lombardy ICU Network	
Marco	Carbonara		MD	Fondazione IRCCS Ca' Granda Osped	Milan, Italy	Patient enrollment anc	COVID-19 Lombardy ICU Network	
Stefania	Crotti		MD	Fondazione IRCCS Ca' Granda Osped	Milan, Italy	Patient enrollment anc	COVID-19 Lombardy ICU Network	
Chiara	Fusaris		MD	Fondazione IRCCS Ca' Granda Osped	Milan, Italy	Patient enrollment anc	COVID-19 Lombardy ICU Network	
Paolo	Properzi		MD	Fondazione IRCCS Ca' Granda Osped	Milan, Italy	Patient enrollment anc	COVID-19 Lombardy ICU Network	
Nicola	Rossi		MD	Fondazione IRCCS Ca' Granda Osped	Milan, Italy	Patient enrollment anc	COVID-19 Lombardy ICU Network	
Paola	Tagliabue		MD	Fondazione IRCCS Ca' Granda Osped	Milan, Italy	Patient enrollment anc	COVID-19 Lombardy ICU Network	
Daniela	Tubiolo		MD	Fondazione IRCCS Ca' Granda Osped	Milan, Italy	Patient enrollment anc	COVID-19 Lombardy ICU Network	
Giuseppe	Maggio		MD	Fondazione IRCCS Policlinico San Ma	Pavia, Italy	Patient enrollment anc	COVID-19 Lombardy ICU Network	
Michele	Pagani		MD	Fondazione IRCCS Policlinico San Ma	Pavia, Italy	Patient enrollment anc	COVID-19 Lombardy ICU Network	
Fabio	Sciutti		MD	Fondazione IRCCS Policlinico San Ma	Pavia, Italy	Patient enrollment anc	COVID-19 Lombardy ICU Network	
Michele	Bertelli		MD	Fondazione Poliambulanza Hospital	Brescia, Italy	Patient enrollment anc	COVID-19 Lombardy ICU Network	
Carmine Rocco	Militano		MD	Fondazione Poliambulanza Hospital	Brescia, Italy	Patient enrollment anc	COVID-19 Lombardy ICU Network	
Alessandro	Morandi		MD	Fondazione Poliambulanza Hospital	Brescia, Italy	Patient enrollment anc	COVID-19 Lombardy ICU Network	
Roberto	Valsecchi		MD	General Hospital "Moriggia Pelascini	Gravedona, Italy	Patient enrollment anc	COVID-19 Lombardy ICU Network	
Giovanni	Albano		MD	Humanitas Gavazzeni	Bergamo, Italy	Patient enrollment anc	COVID-19 Lombardy ICU Network	
Enrico	Barbara		MD	Humanitas Mater Domini Hospital	Castellanza, Italy	Patient enrollment anc	COVID-19 Lombardy ICU Network	
Andrea	Ballotta		MD	IRCCS Centro Cardiologico Monzino	Milan, Italy	Patient enrollment anc	COVID-19 Lombardy ICU Network	
Elena	Costantini		MD	IRCCS Humanitas Clinical and Resear	Rozzano, Italy	Patient enrollment anc	COVID-19 Lombardy ICU Network	
Massimiliano	Greco		MD	IRCCS Humanitas Clinical and Resear	Rozzano, Italy	Patient enrollment anc	COVID-19 Lombardy ICU Network	
Paolo	Perazzo		MD	IRCCS Istituto Ortopedico Galeazzi	Milan, Italy	Patient enrollment anc	COVID-19 Lombardy ICU Network	
Roberto	Ceriani		MD	IRCCS Multimedica	Sesto San Giovanni, Italy	Patient enrollment anc	COVID-19 Lombardy ICU Network	
Marco	Ranucci		MD	IRCCS Policlinico San Donato	Milan, Italy	Patient enrollment anc	COVID-19 Lombardy ICU Network	
Marco	Resta		MD	IRCCS Policlinico San Donato	Milan, Italy	Patient enrollment anc	COVID-19 Lombardy ICU Network	
Maria Rosa	Calvi		MD	IRCCS San Raffaele Scientific Institute	Milan, Italy	Patient enrollment anc	COVID-19 Lombardy ICU Network	
Sergio	Colombo		MD	IRCCS San Raffaele Scientific Institute	Milan, Italy	Patient enrollment anc	COVID-19 Lombardy ICU Network	
Antonio	Dell'Acqua		MD	IRCCS San Raffaele Scientific Institute	Milan, Italy	Patient enrollment anc	COVID-19 Lombardy ICU Network	
Francesco	Marino		MD	Istituto Clinico Beato Matteo	Vigevano, Italy	Patient enrollment anc	COVID-19 Lombardy ICU Network	
Pietro	Sebastiano		MD	Istituto Clinico Città di Brescia	Brescia, Italy	Patient enrollment anc	COVID-19 Lombardy ICU Network	
Giuseppe	Sala		MD	Istituto Clinico Città Studi	Milan, Italy	Patient enrollment anc	COVID-19 Lombardy ICU Network	
Nicolangela	Belgiorno		MD	Istituto Clinico San Rocco	Ome, Italy	Patient enrollment anc	COVID-19 Lombardy ICU Network	

Supplemental Online Content: Nonauthor Collaborators
*First name, last name, and suffix (if applicable) are required and will appear in PubMed.

***First Name and Middle Initial(s)**	***Last Name**	***Suffix (eg, Jr, III)**	Academic Degrees	Institution	Location (city, state/province, country)	Role or Contribution, eg, chair, principal investigator	Group (if more than 1 Group listed in the byline) and/or Subgroup (eg, Steering Committee)	
Rinaldo	Cosio		MD	Istituto Clinico Sant'Anna	Brescia, Italy	Patient enrollment and	COVID-19 Lombardy ICU Network	
Luca	Guatteri		MD	Ospedale "Sacra Famiglia" Fatebenet	Erba, Italy	Patient enrollment and	COVID-19 Lombardy ICU Network	
Uberto	Viola		MD	Ospedale "San Pellegrino" Gruppo M	Castiglione delle Stiviere, I	Patient enrollment and	COVID-19 Lombardy ICU Network	
Marco	Galletti		MD	Ospedale Valduce	Como, Italy	Patient enrollment and	COVID-19 Lombardy ICU Network	
Vincenzo	Primerano		MD	Policlinico di Monza	Monza, Italy	Patient enrollment and	COVID-19 Lombardy ICU Network	
Enrico	Visetti		MD	Policlinico di Monza	Monza, Italy	Patient enrollment and	COVID-19 Lombardy ICU Network	
Matteo	Giacomini		MD	Policlinico San Marco	Zingonia, Italy	Patient enrollment and	COVID-19 Lombardy ICU Network	
Armando	Alborghetti		MD	Policlinico San Pietro	Ponte San Pietro, Italy	Patient enrollment and	COVID-19 Lombardy ICU Network	
Alberto	Bertazzoli		MD	Spedali Civili University Hospital	Brescia, Italy	Patient enrollment and	COVID-19 Lombardy ICU Network	
Michele	Bertoni		MD	Spedali Civili University Hospital	Brescia, Italy	Patient enrollment and	COVID-19 Lombardy ICU Network	
Tommaso	Mauri		MD	Università degli Studi di Milano	Milan, Italy	Patient enrollment and	COVID-19 Lombardy ICU Network	
Giada	Prato		MD	Università degli Studi di Milano	Milan, Italy	Patient enrollment and	COVID-19 Lombardy ICU Network	
Nino	Stocchetti		MD	Università degli Studi di Milano	Milan, Italy	Patient enrollment and	COVID-19 Lombardy ICU Network	
Luigi	Vivona		MD	Università degli Studi di Milano	Milan, Italy	Patient enrollment and	COVID-19 Lombardy ICU Network	
Giulia	Waccher		MD	Università degli Studi di Milano	Milan, Italy	Patient enrollment and	COVID-19 Lombardy ICU Network	
Matteo	Filippini		MD	University of Brescia	Brescia, Italy	Patient enrollment and	COVID-19 Lombardy ICU Network	
Nicola	Latronico		MD	University of Brescia	Brescia, Italy	Patient enrollment and	COVID-19 Lombardy ICU Network	
Marco	Giani		MD	University of Milano-Bicocca	Monza, Italy	Patient enrollment and	COVID-19 Lombardy ICU Network	
Matteo	Pozzi		MD	University of Milano-Bicocca	Monza, Italy	Patient enrollment and	COVID-19 Lombardy ICU Network	
